# Yeast cell wall extracts from *Saccharomyces cerevisiae* varying in structure and composition differentially shape the innate immunity and mucosal tissue responses of the intestine of zebrafish (*Danio rerio*)

**DOI:** 10.3389/fimmu.2023.1158390

**Published:** 2023-05-25

**Authors:** Mark Rawling, Marion Schiavone, Emmanuelle Apper, Daniel L. Merrifield, Mathieu Castex, Eric Leclercq, Andrew Foey

**Affiliations:** ^1^ Aquatic Animal Nutrition and Health Research Group, School of Biological, Plymouth University, Plymouth, United Kingdom; ^2^ Lallemand SAS, Blagnac, France

**Keywords:** α-mannans, yeast cell wall polysaccharides, Bioactivities, zebrafish, β-1,3-glucans

## Abstract

With the rising awareness of antimicrobial resistance, the development and use of functional feed additives (FFAs) as an alternative prophylactic approach to improve animal health and performance is increasing. Although the FFAs from yeasts are widely used in animal and human pharma applications already, the success of future candidates resides in linking their structural functional properties to their efficacy *in vivo*. Herein, this study aimed to characterise the biochemical and molecular properties of four proprietary yeast cell wall extracts from *S. cerevisiae* in relation to their potential effect on the intestinal immune responses when given orally. Dietary supplementation of the YCW fractions identified that the α-mannan content was a potent driver of mucus cell and intraepithelial lymphocyte hyperplasia within the intestinal mucosal tissue. Furthermore, the differences in α-mannan and β-1,3-glucans chain lengths of each YCW fraction affected their capacity to be recognised by different PRRs. As a result, this affected the downstream signalling and shaping of the innate cytokine milieu to elicit the preferential mobilisation of effector T-helper cell subsets namely Th17, Th1, Tr1 and FoxP3^+^-Tregs. Together these findings demonstrate the importance of characterising the molecular and biochemical properties of YCW fractions when assessing and concluding their immune potential. Additionally, this study offers novel perspectives in the development specific YCW fractions derived from *S. cerievisae* for use in precision animal feeds.

## 1 Introduction

The development of nutritional solutions to improve the health and welfare of aquatic and terrestrial farmed species is at the forefront of contemporary research interests. Indeed, the desire to “prevent rather than cure”, and to minimize the use of antibiotics, are driving the development of functional feed additives (FFAs) technologies such as prebiotics, probiotics and postbiotics to improve animal health and performance. Further success in the implementation of FFA technologies in precision animal feeds reside in the finer characterisation of their specific functional properties *in vivo*. In particular, understanding the interaction between mucosa-associated lymphoid tissues (MALT) and the FFA is critical.

The intestinal environment is largely exposed to self, commensal and potentially pathogenic non-self antigens that are constantly screened and processed by the diverse immune cells of the gut-associated lymphoid tissue (GALT), suggesting a central role of GALT in intestinal immune response during homeostasis (1–3). Accordingly, the gastrointestinal system contains substantial amounts of organised lymphoid tissues with large populations of scattered innate and adaptive effector immune cells. Indeed, in humans nearly 70% of the immune system is housed in the gastrointestinal tract (GIT) where it interacts with gastrointestinal function in a dynamic manner (4); for instance, directing the immune response towards the luminal contents allowing for either tolerance or degradation of luminal antigens. The immune mechanisms implicated in this action are very complex and are governed by both innate and adaptive immunity. Innate immunity is an immediate response that is indispensable before a specific adaptive immunity is mobilised, that requires a longer period to be efficacious (5). In teleost fish immune system, most of the innate immune functions are similar to mammals but there are some differences in the structure of GALT (6–8). Although there is no clear evidence that teleost fish have Peyer’s patches and mesenteric lymph nodes, the presence of innate immune cells, TCRγδ^+^ T-cells and B1-B cells in and under the gut epithelium across the entire intestinal tract (9–13) make teleost fish a valid comparative model to higher vertebrates.

Zebrafish (*Danio rerio*) is a good comparative model to investigate the mode of action of functional feed compounds on mucosal immune responses due the high presence (~70%) of human orthologous genes including genes associated with diseases (14). Indeed, counterparts of mammalian pathogen recognition receptors (PRRs), such as Toll-like receptors (TLRs) and nucleotide-binding oligomerization domain-like receptors (NLRs), as well as downstream signalling components, have been demonstrated to play important roles in zebrafish host defence (15). Furthermore, upon comparison of the zebrafish GIT with that of the stickleback, mouse and human, Lickwar and colleagues (6) reported a core set of genes that were highly expressed in all vertebrate intestinal epithelial cells (IECs) and displayed functional conservation across a broad range of IEC biology involved in lipid transport, metabolism, response to microbes and inflammation. Moreover, from a cellular level zebrafish have an abundance of innate immune cells that interplay with γδT-cells in the recognition and processing of antigens that cross the luminal barrier of the intestine (16).

Within the last decades, FFAs derived from *Saccharomyces cerevisiae* yeast have become among the most widely used functional ingredients in animal and human feed. *S. cerevisae* yeast cell wall (YCW) holds an important position among multifarious prebiotics and have been implicated to suppress the adhesion of potentially pathogenic bacteria *via* GIT agglutination (17, 18) and exert beneficial effects on growth performance, intestinal structure and immunity as well as gut microflora in farmed and companion animals (19–21). *S. cerevisae* is composed of 90% of polysaccharides typically consisting of 30 to 40% mannans and 50 to 60% of highly branched β-1,3/1,6-glucans. Mannans, which constitute the outermost layer of the cell wall, have a complex structure that includes α-1,6-1,3-1,2-mannosyl linkages and are covalently attached to the cell wall proteins *via* either asparagine (N-linked mannans) or serine/threonine (O-linked mannan) residue to form mannoproteins (22). β-glucans, which are found beneath the mannans layer, consist of long β-1,3-glucose polymer with β-1,6-glucan side branches (23). Mannans and β-glucans are documented to be recognised by different PRRs such as C-type lectins including Dectin 1, 2 and 3, DC-SIGN and Mannose-binding receptor; and Toll-like receptors (24).

YCWs are highly diverse in their biochemical composition, molecular structure and cell wall architecture which can vary with the yeast species and strain, the growth conditions of the yeast and the YCW production process (25). Such diversity in the biophysical properties of the YCW can be expected to elicit distinct host-immune responses but this has, to date, largely been under-evaluated. Therefore, contemporary research on the use of yeast-based technologies requires more effort to link the structural characteristics of YCW with their subsequent functional effects *in vivo*.

The current investigation aimed at characterising the biochemical and molecular properties of four proprietary *S. cerevisiae* YCW fractions of discrete origins in relation to their potential effect on the intestinal immune responses when administered orally. Our hypothesis was that the immune properties of a given *S. cerevisiae* YCW varies with its structural and molecular characteristics which, to the authors’ knowledge, has not been previously addressed using a purposely designed comparative study. This information will facilitate the development of specific YCW fractions eliciting targeted immune functionalities for use in precision animal nutrition.

## 2 Materials and methods

### 2.1 Experimental system and fish

Experimentation was carried out at the Aquaculture and Fish Nutrition Research Aquarium, University of Plymouth (Plymouth, UK) within an indoor freshwater recirculated aquaculture system (RAS) equipped with mechanical and biological filtration, aeration, photo-and-thermo control. The RAS system consisted of 15 rectangular fibreglass tanks (15 litre/tank) each set with a water flow rate of 15 litre/hr. Wild type zebrafish stocks were originally sourced from the European zebrafish resource centre (EZRC, Germany); F2 generations were bred from the original stock and used in the experiments. For this trial, 375 fish were randomly distributed into the experimental system (25 fish/tank, initial mean body-weight (BW_i_) = 0.80 ± 0.02 g) at the beginning of the trial. During the trial, fish were kept under a constant 12:12 hr light:dark photoperiod and water quality parameters were maintained within a suitable range for zebrafish as follows: water temperature = 25 ± 0.5 °C, pH = 6.8 to 7.5, dissolved oxygen = 7.5 to 8.0 mg/l, total ammonia = 0.04 to 0.08 mg/l, nitrite = 0.02 to 0.06 mg/l and nitrate = 54 to 58 mg/l. Approval was given by the University of Plymouth’s animal ethical review board under number: ETHICS-32-2019.

### 2.2 Experimental diets

A basal diet was formulated (36% crude protein and 8% crude lipid) using feed formulation software (Feedsoft^®^) to meet the known nutritional requirements of cyprinids (26). The test diets were produced by supplementing, prior to cold press, the mash basal diet with one of four YCW fractions (YCW1, 2, 3 or 4; Lallemand SAS, Blagnac, France) at 2.0 kg/T of feed each (**Table 1**). Each YCW fraction was provided in the form of a pure, dry (moisture ≤ 8%), fine, light tan powder. The diets were produced by mechanically stirring the ingredients into a homogenous mixture using a Hobart food mixer (Hobart Food Equipment, Australia, model no: HL1400 — 10STDA mixer). Warm water was added to reach a consistency suitable for cold press to form 1 mm pellets (PTM Extruder system, model P6, Italy). Each diet was then dried, ground and sieved to isolate pellets of ϕ600-800 µm for the trial. The nutritional profile of the diets was determined according to AOAC protocols (27).

**Table 1:** Formulation (g/kg) and proximate composition (%) of the experimental diets.

			Control diet		YCW diets	
Formulation (g/kg)						
Soybean meal dehulled^1^			150.0		150.0	
Soy Protein Concentrate (SPC60)			154.0		154.0	
Sunflower meal			220.0		220.0	
Wheat meal			166.4		166.4	
Fabameal			220.0		220.0	
Rapeseed oil			59.2		59.2	
Vitamin and mineral premix^2^			10.0		10.0	
Lysine HCL^3^			9.3		9.3	
DL-methionine^3^			8.3		8.3	
Gelatin^3^			10.0		10.0	
Yeast cell wall fraction^4^					2.0	
Proximate composition	Control		YCW1	YCW2	YCW3	YCW4
Dry matter (DM; %)	94.8 ± 0.9		94.4 ± 0.5	94.7 ± 0.4	94.7 ± 0.1	94.5 ± 0.3
Crude protein (% DM)	36.4 ± 0.6		36.4 ± 0.5	36.2 ± 0.1	36.3 ± 0.4	36.5 ± 0.1
Crude lipid (% DM)	6.4 ± 0.4		6.5 ± 0.2	6.6 ± 0.2	6.8 ± 0.3	6.7 ± 0.2
Ash (% DM)	4.3 ± 0.2		4.2 ± 0.1	4.2 ± 0.1	3.6 ± 0.5	3.9 ± 0.4

^1^HP-110, Hamlet Protein A/S (Horsens, Denmark): crude protein 57.5%; ash 6.8%; moisture 6.5%; lipid 2.5%).

^2^Premier Nutrition (Rugeley, UK): Calcium 12.1%, magnesium 1.6%, phosphorous 0.5%, vit A 1.0µg/kg, vit D3 0.1 µg/kg, vit E (as alpha tocopherol acetate) 7,000 mg/kg, copper (as cupric sulphate) 250.0 mg/kg, ash 78.7%.

^3^Sigma-Aldrich (Poole, UK).

^4^Lallemand SAS (Blagnac, France).

### 2.3 Experimental design and feeding

The trial lasted 5 weeks during which zebrafish were fed one of five diets in triplicate tanks: 1) Control (basal diet), 2) YCW1, 3) YCW2, 4) YCW3, and 5) YCW4. Fish were hand-fed at 4.0% biomass per day distributed in three equal meals (0900, 1300 and 1600 h). Biomass per tank was estimated daily based on predicted growth rate and adjusted weekly by bulk-weighing each tank following a 24 h starvation period.

### 2.4 Sampling schedule

At the end of the trial, 9 fish per tank (27 fish/treatment) were randomly netted and euthanized following UK Home Office schedule 1 procedures prior to sampling under a microdissection microscope. Among these, 3 fish per tank (9 fish/treatment) were dissected for histology as follows. Posterior intestinal (PI) samples were excised and digesta was removed using phosphate buffer saline (pH 7.2, Sigma Aldrich, UK), fixed in 10% neutral buffered formalin (pH 7.0; Sigma Aldrich, UK) kept at 4°C for 24 h followed by long-term storage in 70% ethanol at room temperature until processing.

The remaining 6 fish per tank were sampled for gene expression analysis as follows. PI samples were excised and stored in 500 µl of RNA later solution (Applied Biosystems, UK) and kept at 4°C for 24 h then at -80°C until processing. For each biological replicate, PI from 3 fish per tank were pooled together giving 2 samples per tank (6 samples/treatment).

### 2.5 Biochemical composition of YCW

The biochemical composition of the YCW samples was determined by the sulfuric acid method according to the protocol of (28), followed by analysis of the released monosaccharides (glucose and mannose) to determine mannans and β-glucans content as the sum of β-1,3 and β-1,6-glucans, respectively. Sugars monosaccharides (mannose and glucose) were analysed by high performance liquid chromatography with an evaporative light scattering detector (Varian-385-LC ELSD). A Rezex™ RCM-Monosaccharide Ca+2 column (300 x 7.8 mm; Phenomenex) was used to separate monosaccharides at 80°C by an isocratic elution for 20 min at 0.6 mL/min with ultrapure water.

### 2.6 Scanning electronic microscopy (SEM) and Atomic Force Microscopy (AFM) imaging of YCW

YCW samples were imaged by SEM on a SEM Quanta 250 FEG FEI at 10 kV. The surface of the YCW was analysed by a Nanowizard III Atomic Force Microscope (AFM; Bruker) after immobilisation of yeast cells by mechanical trapping into polydimethylsiloxane (PDMS) stamps (29). Single-molecule force spectroscopy experiments were carried out using MLCT probes (Bruker) with a 0.002 N.m^-1^ spring constant, that were functionalised with concanavalin A (ConA; Sigma-Aldrich) and a mouse monoclonal anti-β-1,3-glucan (Biosupplies) as described by Schiavone and colleagues (30). Force mapping were obtained by recording 1024 force-distance curves on each cell (at least 8 cells were analysed per YCW). The binding was calculated as the percentage of retract curves presenting adhesion events on the total of force-distance curves analysed (n=8192). Force curves with adhesion force <20 pN or a distance tip-sample (rupture length) of zero were considered as non-adhesive curves. All the curves were analysed with JPK Data Processing software (Bruker-JPK Instruments). The distance required to break the interaction between ConA or anti-β-1,3-glucan at the apex of the AFM tip and the α-mannan or β-1,3-glucan respectively at the yeast cell surface was measured and used to determine the contour length of the corresponding polysaccharides.

To characterize the stretching of polysaccharides at the surface of the cell, elongation forces on the force-distance curves were analysed with the worm-like chain (WLC) model introduced in Bustamante and colleagues (31), which describes the polymer as a curved filament. The contour length from this model represents the length of the polysaccharide stretched or unfolded. All adhesion and contour length values were considered for the histograms, which were generated using OriginPro version 2020 (OriginLab, Northampton, MA) and fitted with a Gaussian curve to obtain the most probable value of the adhesion force and the length of α-mannan or β-1,3-glucan unfolded for each YCW.

### 2.7 Intestinal morphometry by light microscopy

Formalin-fixed PI samples were dehydrated in a gradient ethanol series (Leica TP1020), embedded in paraffin wax for longitudinal sectioning at 3 µm thickness (Leica RM2235 microtome). Multiple consecutive sections for each sample were stained with haematoxylin and eosin (H&E) to assess muscularis thickness (MT), mucosal fold height (VL), lamina propria width (LPW), and intraepithelial leukocyte abundance (IELs) per 100 enterocytes after Rawling and colleagues (32). Alcian blue-period acid Schiff (AB-PAS) stain was used to assess goblet cell density (GCD) and goblet cell mucin chemotype after Rawling and colleagues (32). Quantitative measurements of each image were taken using Image ‘J’ 1.47v software (National Institutes of Health, USA).

### 2.8 Transcriptomic analysis

RNA extraction and cDNA synthesis were performed according to Rawling and colleagues (28). Briefly, 20 mg PI sample was transferred into a microcentrifuge tube containing 1 ml of TRI reagent and homogenised using ceramic beads for 40 sec on the FastPrep-24 5^G^ machine following the manufacturer’s instructions (MP Biomedical, EU). A 200 µl volume of chloroform was added, mixed and centrifuged (12,000 x *g*; 15 min; 4°C). The supernatant was removed, and 500 µl of isopropanol was added and centrifuged (14,000 x *g*; 15 min; 4°C), to precipitate the RNA. RNA was cleaned using 70% molecular grade ethanol. Total RNA was dissolved in diethylpyrocarbonate (DEPC) treated water. Any contaminating genomic DNA was removed using the DNase max kit following manufacturer’s instructions (Qiagen, UK). The concentration and quality of RNA in each sample was determined by measuring 260/280 nm and 260/230 absorbance ratios (NanoDrop Technologies, Wilmigton, USA). The integrity of RNA was confirmed by running samples on a 1% agarose gel and RNA samples were stored at -80°C. A total amount of 1 µg of RNA was used for cDNA synthesis, employing iScript cDNA synthesis kit (Bio-Rad, UK). The reaction was placed at 25°C for 5 min, then 46°C for 20 min and inactivated at 95°C for 1 min. The iScript cDNA synthesis kit contains a combination of oligo dTs and random hexamers to work with a wide variety of targets.

The real-time PCR assay was performed according to Rawling and colleagues (32). Briefly, PCR reactions were set on a 384 well plate in duplicate per sample. Each reaction was mixed with 2 µl of diluted (1/10) cDNA and 5.5 µl 2 x concentrated iQ™ SYBR Green Supermix (Bio-Rad), 0.3 µM forward primer and 0.3 µM reverse primer. The primer used and their sequences are presented in **Table 1**. Florescence monitoring occurred at the end of each cycle and an additional dissociation curve analysis was performed showing a single peak per sample. *Elf1-α* and *metap1* were used as reference genes in each sample to standardise the results by eliminating variation in mRNA and cDNA quantity and quality (33). The stability of *elf1-α and metap1* as reference genes were confirmed by an expression stability value ‘M’ generated by the geNorm™ software. Modification of gene expression was presented with respect to the controls being sampled at the same time as the treatment. PCR efficiencies for primer sets were determined using 10-fold serial dilutions of cDNA and presented using the equation E (PCR efficiency) = 10(-1/slope; **Table 2** and **Supplementary Table 3**). The expression of target genes was displayed as fold change (FC (Log2) and were calculated based on Ct deviation (δCt) of the unknown sample versus a control sample and expressed in comparison to the reference genes *elf1-α* and *metap1*.

**Table 2:** Primer pair sequences, gene name abbreviations, annealing temperature (Aneal Tm in °C), amplicon size (bp) and primer efficiency (Eff) for genes used for real-time PCR.

Gene	Functional annotation	Accession number	Primer sequence (5’-3’)	Efficiency
Reference genes				
*elf1*α	elongation factor 1 alpha	L23807.1	F-AGATGCCGCCATTGTTGAGA R-CTCTTGGTCGCTTTGCTGTG	2.1
*metap1*	methionyl aminopeptidase 1	NM_001025165.2	F-GACGAGGGAGCCAAGAGATT R-TCTGTGAAGCCTGGTATCCG	2.1
MAMP recognition				
*tlr2*	toll-like receptor 2	NM_212812.1	F-GTCCCATCGGTTCAGTCTCTT R-GTTTCAGGGTGGGAGACATCT	2.1
*tlr4bb*	toll-like receptor 4b, duplicate b	NM_212813.2	F-TCAACCAGAGCTGACACATCT R-CAGAAAGGTTCATGGGCAACTT	2.2
*marco*	macrophage receptor with collagenous structure	KJ955494.1	F-CTGGGAGGAAGGGAGATTCAG R-ACCAGTCCTGCCATCTTGAC	1.9
Signal transduction				
*myd88*	myeloid differentiation factor 88	NM_212814.2	F-GACTGACACCTGAGACCTTTGA R-TCGGTGTGTTCCAACTGTTTG	1.9
*traf6*	Tumor necrosis factor (TNF) receptor-associated factor 6	NM_212814.2	F-GAGAAGGAGAGGGAGTCGTTTC R-TGCTGGTCAGGAGGCATACT	1.9
*tollip1*	toll interacting protein (tollip), transcript variant 1	NM_207061.2	F-CCTGTGGTTCTGATGCCTACA R-GGCACCACACCCTGATTATACA	1.8
Transcription factors				
*nfκB*	Nuclear factor kappa B	NM_001001840.3	F-TGCTGACACTCACCCATCTG R-GACCACCACTCAACTGATAGC	1.8
*stat4*	signal transducer and activator of transcription 4	NM_001004510.1	F-AGTCCACTGGCTGTCTGTCT R-CAGCGGACCCTCATTTCCTT	2.1
*rorc*	related orphan receptor c	EF107094.1	F-CTGAAGAGATCCGTGTCTACCA R-TGGCAAACTCCACCACATACT	2.1
*stat5a*	signal transducer and activator of transcription 5a	NM_194387.2	F-ACAAGTAGTGCCAGAGTTTGC R-GCTGGAGATGATGCTACATGGT	1.9
*foxp3a*	forkhead box P3a	NM_001329567.1	F-TGCGTGTTGAAGGAAGGAAAG R-GGTGCCATCCAGTCCATATCA	2.2
Cytokines				
*tnfα*	tumor necrosis factor alpha	NM_212859.2	F-CCATAAGACCCAGGGCAATCA R-GGCAGCCTTGGAAGTGAAATTG	1.9
*ifnγ (ifng)*	interferon gamma 1	NM_212864.1	F-CCCATCTTCCTGCGAATCCT R-GCTTCATCCACGCTGTCATTC	2.1
*il17a*	interleukin 17a/f1	NM_001020787.1	F-ACATAACGAGAGCCTGTATCCT R-CCTCAACGCCGTCTATCAGA	2.2
*il10*	interleukin 10	NM_001020785.2	F-CCCTATGGATGTCACGTCATG R-TCCCGCTTGAGTTCCTGAAA	2.2
*tgfβ*	transforming growth factor, beta 3	NM_194386.2	F-AGGACAACACTGAGACTGAGTA R-GCAGTAGGGCAGGTCATTGT	2.0

### 2.9 Statistical analysis

All statistical analyses were carried out using R version 3.4.1 (34). Rt-qPCR data were analysed using the permutation after Ohmel (35). Redundancy analysis of gene expression profiles for cytokines was performed using vegan package in R (36). All other data were assessed by one-way ANOVA tests with Tukey HSD *post-hoc* analysis where differences occurred. Data are presented as mean ± standard deviation (SD). Gene expression data showing comparisons with the control group are presented as means ± standard error of the mean (SEM; fold change (Log2)). AFM data represent cumulative results of all experiments performed and are shown as means ± S.D. The significative differences between YCW were determined by one-way analysis of variance (ANOVA) test with Tukey HSD *post-hoc* analysis. For all analysis, significance was accepted at P < 0.05.

## 3 Results

### 3.1 YCWs imaging, composition and functional characterisation

**Figure 1:**
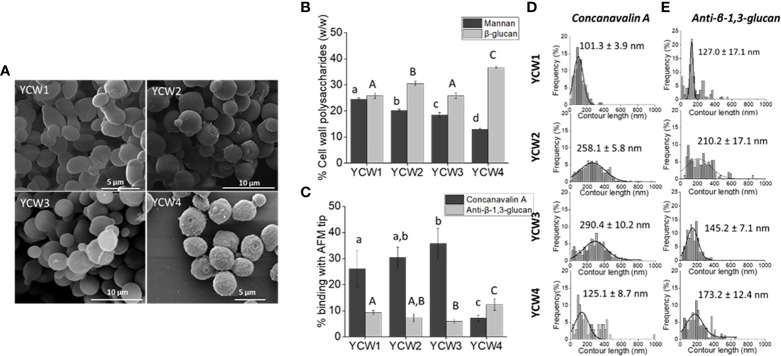
YCW fractions are characterised by differences in cells surface structures and composition. **(A)** Scanning electron microscopy images of each YCW fraction. **(B)** YCW biochemical composition of bioactive compounds, as the total percentage of α-mannans and total β-glucans for β-1,3 and β-1,6-glucans present in each YCW fraction. **(C)** Occurrence of binding with Concanavalin A and anti-β-1,3-glucan tip at the surface of each YCW fraction. Frequency distribution plots of **(D)** mannan-chains and **(E)** β-1,3-glucans contour length mapped at the surface of each YCW fraction using an AFM tip functionalised with Concanavalin A and anti-β-1,3-glucan, respectively; shown with mean values. All contour length values are plotted. Results are expressed as mean ± standard deviation value. Different letters indicate significative differences (One-way ANOVA, p < 0.05) between YCWs within each separate parameter as distinguished using small or large letters.

To assess the biochemical and nanomechanical properties of each yeast cell wall fraction several assays were performed. Scanning electronic microscopy imaging revealed well round-shaped YCWs with a preserved cellular integrity, i.e. without apparent breakage (**Figure 1A**). YCW fraction 4 presented a rougher cell wall surface due to the removal of the outer layer of the cell wall.

The biochemical composition in bioactive compounds, i.e. mannans and β-glucans, was significantly different between each YCWs (**Figure 1B**). YCW1 had the highest content of mannans (24.5%) and YCW group 4 the lowest (13%). Inversely, YCW1 had the lowest and YCW4 the highest β-glucans content (25.8% and 36.7%, respectively).

Yeast cell surface was mapped using Concanavalin A (ConA) and anti-β-1,3-glucan functionalised AFM tips. The occurrence of binding events between α-mannan and ConA-functionalised AFM tips, as well as between β-1,3-glucan and anti-β-1,3-glucan tip at the surface of YCW1 was significantly different from YCW3 and 4 (**Figure 1C**; *p* < 0.05). Over the total number of force curves analysed, 26.1%, 30.4%, 35.8% and 7.1% of interactions with ConA were detected respectively for YCW1, 2, 3 and 4. While the proportion of total mannans and occurrence of binding with ConA were consistent for YCW1 (24.5% and 26.1%, respectively), the surface of YCW2 and 3 interacted more frequently with ConA compared to YCW1 despite their significantly lower total mannan content (*p* < 0.05; **Figure 1B**). Finally, YCW4 had both the lowest levels of mannans and binding with ConA but these showed contrasted levels (13% and 7.1%, respectively). The mechanical properties of the cell wall α-mannans at the cell surface of the yeast were measured by rupture distance that corresponds to the distance at which the binding of the polysaccharide with the AFM-tip ConA was broken. The results suggest that the elongation forces on the cell surfaces of all YCW fractions were better described by the WLC model, which resulted in contour lengths as large as 600 nm in YCW fraction 2 and 3 and up to 400 nm in YCW fractions 1 and 4. As a result it could be proposed that in all YCW fractions the entire mannoproteins and not solely the α-mannan chains were stretched out from the cells wall and there were detectable differences in the length of these polysaccharide structures (**Figure 1D**). The mannans contour length averaged 258.1 ± 5.8 nm and 290.4 ± 10.2 nm for YCW2 and 3, respectively, which was approximately double to that of YCW1 and 4 (**Figure 1D**).

Mapping of β-1,3-glucans at the surface of YCW using a monoclonal mouse anti-β-1,3-glucan measured 9.3%, 7.3%, 5.9% and 12.4% of interactions for YCW1, 2, 3 and 4, respectively (**Figure 1C**); overall showing a lower ratio of interactions to composition compared to that of mannans - ConA. This observation was expected as the AFM technique allows to map only β-1,3-glucans accessible at surface of the cell, while the biochemical analysis quantifies the amount of total β-glucans without distinguishing β-1,3-glucan from β-1,6-glucan. Similar to the detection method used for α-mannans, β-1,3-glucans stretching was described using the WLC model, indicating a semi-flexible conformation of this polysaccharide, in accordance with the triple helix structure of β-1,3-glucans. The contour length of β-1,3-glucans chains from YCW 1 and 3 were distributed over a narrow range of 10 to 400 nm compared to YCW2 and 4 for which values of up to approximately 600 nm were measured. The average β-1,3-glucan contour lengths were 127.0 ± 17.1 nm and 145.2 ± 7.1 nm for YCW1 and 3 which were lower than the values measured for YCWs 2 and 4 (**Figure 1E**).

### 3.2 Intestinal morphometry reveals strengthening of intestinal barrier

The PI of zebrafish fed the different YCWs for 5 weeks were closely examined as the intestine is a primary site of endocytic and pinocytotic activity for antigen sensing and uptake (Romboult et al., 2011[28]). The sampled intestine revealed no signs of enteritis or necrosis-like pathologies; there was no effect of feeding the YCW fractions on muscularis thickness and mucosal fold length, but there was a significant effect of YCW group 2 on lamina propria width which was lower compared to all other treatments (**Supplementary Figure 1** and **Table 1**).

**Figure 2:**
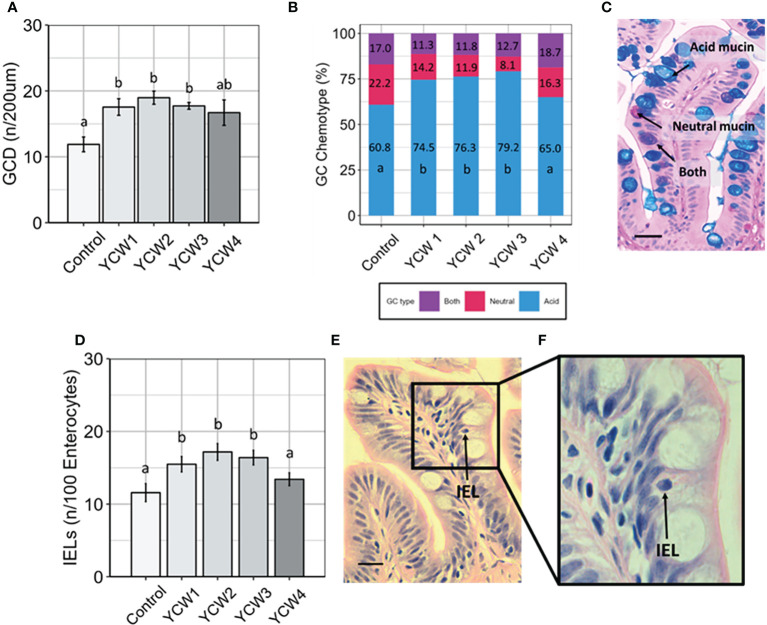
Fortification of the intestinal barrier by elevation of goblet cell density, acidomucin secreting cells and intra-epithelial leucocytes (IELs) when fed different yeast cell fractions. **(A)** Goblet cell density (GCD, n/200 µm of intestinal villi; mean ± SD with 9 fish/group assessed; **(B)** Relative abundance of goblet cell (GC) chemotypes as identified using Ab-PAS differential staining; **(C)** Example of Ab-PAS stained villi showing acidic (blue), neutral (pink), both (purple) mucins (scale bar= 10 µm). **(D)** Abundance of IELs (n/100 enterocytes; mean ± SD with 9 fish/group assessed; **(E, F)** Example of H&E stained mucosal fold showing an intraepithelial leukocyte (black arrow; Figure E, scale bar 10 = µm; **(F)** Different letters indicate significant differences between treatments (p < 0.05).

Compared to the control group, goblet cell density (GCD; **Figure 2A**) was significantly higher in YCW groups 1, 2 and 3 (+32.4%, +37.4% and +32.8%, respectively). Further, there were significant elevations in the prevalence acidomucin goblet cells (**Figures 2B, C**) in YCW 1, 2 and 3 fed groups (+13.7 pp, +15.5 pp, +18.4 pp, respectively) compared to the control (**Figure 2A**). Similar to the results observed with GCD, IEL abundance (**Figures 2D–F**) was significantly elevated in YCW groups 1, 2 and 3 compared to control (+25.2%; +29.2% and +32.6% respectively; **Figure 2D**) which was not observed in YCW group 4.

### 3.3 Different YCW fractions differentially modulate of innate immune PRRs and signal transduction

**Figure 3:**
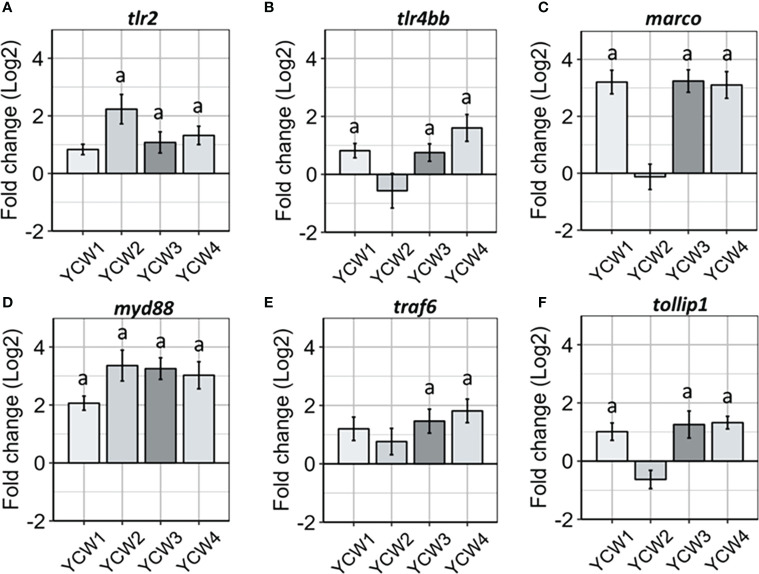
Oral administration of different YCW fractions differentially modulate the gene expression of host PRRs and innate immune signal transduction markers. Total RNA was isolated from the posterior intestine of zebrafish and gene expression of **(A–C)** PRR (*tlr2, tlr4bb* and *marco*) and **(D–F)** signal transduction markers (*myd88, traf6* and *tollip 1*) were evaluated by RT-qPCR. Data expressed as fold-change (Log2) relative to the control and shown as mean ± SEM (n = 6 per group); Presence of a letter highlight significant differences to the control and different letters highlight significant differences between treatments (p < 0.05).

Innate immune cells express different classes of innate immune PRRs, including scavenger receptors (SRs) and the microbe sensing toll-like receptors (TLRs). Indeed, members from both families have been implicated for the detection of microbial associated molecular patterns (MAMPs) derived from the yeasts such as *Saccharomyces cerevisiae*. Compared to the control, there was a significant elevation in the expression of *tlr2* in YCW groups 2, 3 and 4 (+84.1%, +58.6% and +64.6% respectively, *p* < 0.05) while the expression of *tlr4bb* was significantly elevated in YCW groups 1 (+74.3%; *p* = 0.006) and 4 (+47.4%; *p* = 0.01) and that of *marco* in YCW groups 1, 3 and 4 (+91.1%; +91.2% and +90.9%; *p* = 0.002). Although not significant, the expression of *tlr4bb* and *marco* was down regulated by 9.1% and 8.3%, respectively in the YCW 2 group compared to the control (**Figures 3B, C**).

Regards to signal transduction markers (**Figure 3B**) and compared to the control; *myd88* expression was significantly upregulated in all four YCW groups (from +74.5% in YCW1 to +93.0% in YCW2 group) while *traf6* was upregulated in the YCW groups 3 and 4 (+71.4%, *p* = 0.01; +77.8%, *p* = 0.002, respectively) and *tollip1* in YCW groups 1, 3 and 4 (+52.5%, *p* = 0.04; +68.2%, *p* = 0.04 and +62.8%, *p* = 0.006, respectively). Notably and although not significant, the expression level for *tollip1* was down regulated by 30.7% in the YCW 2 group compared to the control (**Figure 3F**).

### 3.4 Transcriptional gene expression markers show distinct profiles for different YCW fractions

**Figure 4:**
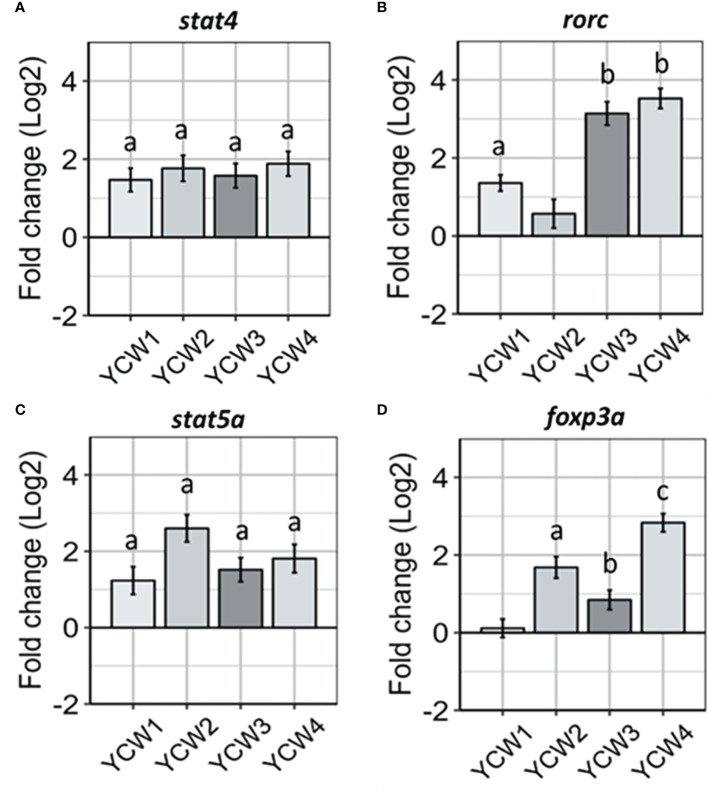
Oral administration of different YCW fractions differentially modulate the expression of host transcription factor. Total RNA was isolated from the posterior intestine of zebrafish and gene expression of **(A)** *stat4*, **(B)** *rorc*, **(C)** *stat5a*, **(D)** and *foxp3a*, were evaluated by RT-qPCR. Data expressed as fold-change (Log2) relative to the control and shown as mean ± SEM (n = 6 per group); Presence of a letter highlight significant differences to the control and different letters highlight significant differences between treatments (*p* < 0.05).

Transcriptional factors for induction of innate immune responses that can prime antigen specific responses are important markers to identify and link cell mediated innate responses with adaptive T-cell responses. In this context the expression level of *stat4*, an important transcription factor for Th1 differentiation through the IL-12 signalling cascade (**Figure 4A**), was up-regulated across all YCW groups compared to the control (from +67.6% in YCW 1 group to +76.3% in YCW 4 group; p < 0.01). In contrast and compared to the control group, the expression of *rorc* gene was significantly elevated in the YCW 1 group (+63.1%, *p* = 0.02) and to a further extent in YCW groups 3 (89.7%; *p* = 0.002) and 4 (+91.9%; *p* = 0.002), but not in YCW group 2 (**Figure 4B**).

Compared to the control (**Figure 4C**), the expression of *stat5a* was significantly elevated in all YCW groups and the highest in the YCW group 2 (+85.1%, *p* = 0.002). In contrast, the expression of *foxp3a* was differentially modulated in the different YCW groups (**Figure 4D**). While YCW group 1 had no apparent effect on *foxp3a* expression level, it was significantly upregulated in YCW groups 3 (47.3%; *p* = 0.03), 2 (+71.4%; *p* = 0.004) and 4 (+86.7%, *p* = 0.002) compared to the control. Notably, the expression of *foxp3a* was significantly down-regulated in the YCW 1 group compared to the YCW groups 2 and 4 (*p* < 0.01).

### 3.5 Different YCW fractions differentially modulate innate immune cytokine profiles

**Figure 5:**
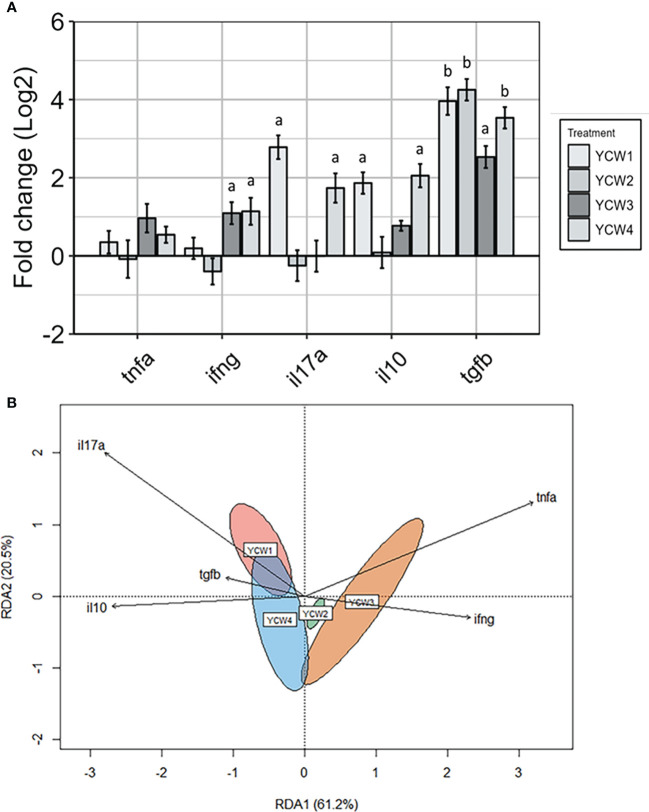
Distinct pro- and anti-inflammatory cytokine profiles in response to oral administration of different YCW fractions. **(A)** Gene expression level of *tnfα, ifnγ, il17a, tgfβ*, and *il10* in the posterior intestine of zebrafish evaluated by RT-qPCR. Data expressed as fold-change (Log2) relative to the control and shown as mean ± SEM (n = 6 per group); Presence of a letter highlight significant differences to the control and different letters highlight significant differences between treatments (p < 0.05). **(B)** Redundancy analysis was a constrained model indicating a significant difference by permutation in the variation of the explanatory variables (YCW group) for each response variable (genes) (r2 = 0.81, p = 0.001). Red ellipse = YCW1, green ellipse = YCW2, orange ellipse = YCW3 and blue ellipse = YCW4. Arrows pointing in the same direction indicate positive correlations, and arrows pointing in opposite directions indicate negative correlations. The arrow length corresponds to the variance explained by the explanatory variable. The first two axes explain 81.7% of the total canonical eigenvalues.

As a measure of immune competency, several well-characterised inflammatory (*il17a*, *tnfα*, *ifnγ*) and anti-inflammatory (*il10* and *tgfβ*) cytokines were analysed (**Figure 5A**). The redundancy analysis (RDA; **Figure 5B**) summarised the main patterns of variation for each response variable (genes) that can be explained by the matrix of explanatory variables (YCW group). The first two axis RDA1 and RDA2 explained 81.7% of the total canonical eigenvalues. The RDA plot revealed strong positive correlations between the explanatory variables for pro-inflammatory cytokines, *ifnγ* and *tnfα*. In the YCW 1 group, the RDA analysis revealed a positive link with the explanatory variable *il17a*. Indeed, the expression of *il17a* in the YCW 1 group was significantly elevated (+87.0%; *p* = 0.002) compared to the control (**Figure 5A**). Moreover, the RDA revealed a positive link with both response variables *il17a* and *ifnγ* in the YCW 4 group. This was supported by significant elevations in the expression of *ifnγ* (+60.3% *p =* 0.03) and *il17a* (+74.8%; *p* = 0.002) in the YCW 4 group compared to the control regime (**Figure 5A**). Likewise, there was a significant elevation in the expression of *ifnγ* (+57.6%; *p* = 0.03) in the YCW 3 group compared to the control and the RDA revealed a positive link with both response variables *ifnγ* and *tnfα* in the YCW 3 group (**Figure 5B**).

The RDA revealed strong positive correlations between the response variables for anti-inflammatory cytokines, *tgfβ* and *il10*. Compared to the control group, there was a significant upregulation in *tgfβ* expression in YCW 1 and 4 groups (+94.4%, *p* = 0.002 and +92.1%; *p* = 0.002, respectively) as well as *il10* (+76.1%; *p* = 0.006 and +79.6%; *p* = 0.004; respectively). In contrast, *tgfβ* but not *il10* was significantly upregulated in YCW groups 2 (94.9%; *p* = 0.002) and 3 (86.1%; *p* = 0.002) compared to the control regime.

## 4 Discussion

Yeast and yeast cell wall fractions derived from *S. cerevisiae* contain functionally conserved molecules acting as microbial associated molecular patterns (MAMPs) that can interact directly or indirectly with pathogens and the host’s immune system (37, 38). The current study assessed the structural-functional relationship of four different proprietary yeast cell wall (YCW) fractions from *S. cerevisiae* towards mucosal tissue response and the intestinal immune response. We documented the discrete biophysical properties of distinct YCWs which were, upon oral administration at the same dosage to zebrafish as a vertebrate model, associated with varying levels of intestinal barrier fortification and distinctive immune molecular signatures from recognition to markers of T-cell differentiation.

Four types of YCW were used with significant differences in mannans and total β-glucan content. Mapping of α-mannans and β-1,3-glucans chains on the cell surface using single-molecule force spectroscopy with functionalised AFM-tips revealed differences in terms of cell coverage and length of these polysaccharides. We highlight two significant YCW characteristics that could be important when considering the potential immune potency of each YCW fraction. Firstly, the proportion of cell coverage and length of α-mannans that decorate the cell surface were different between yeast cell wall fractions. Secondly, the binding of anti-β (1, 3) glucan tip with the cell surface was different between YCW, indicating a difference in the accessibility of β-1,3-glucan at the cell surface which could drive differential recognition by immune host cells and in turn affect their capacity to elicit protection from pathogenic and non-pathogenic agents. The highest cell surface coverage was displayed by YCW4, that was significantly higher compared to all other YCW fractions. Interestingly SEM imaging revealed that YCW4 displayed a rougher outer layer suggesting a more exposed layer and so potential higher exposure of the β-glucan layer. Finally, the mechanical properties of the cell wall β-glucans were studied with a focus on β-1,3-glucans as the AFM-tip only bound to this polysaccharide structure. Elongation forces were described with the WLC model for calculation of the β-1,3 glucan contour lengths, which correspond to the length of the polysaccharides completely unfolded by the AFM tip. The length of β-1,3 glucan structures on the cell surface were different between each YCW fraction; with the highest contour length displayed by YCW2 and being almost double that of YCW1. These apparent structural disparities between each YCW fraction are expected to be due to strain specificities and environmental adaptation to the conditions experienced by the yeast during its propagation. Differences in β-1,3-glucans structure could lead to different response *in vivo* as β-1,3-glucans are classified as biological response modifiers, where the most bioactive β-glucans contain 1,6-linked side chains branching off from the more extended β-1,3-glucan backbone and are referred to as β-1,3/1,6-glucans (39).

To address whether the different structures of the YCW can interact differently with GALT the zebrafish was used as a good comparative model (40) First, the mucosal tissue response was characterised. The intestine represents a major portal of entry for parasites, bacteria and viruses against which goblet cell hyperplasia and subsequent changes in mucin composition constitute an important protective measure against pathogen adherence and translocation across the intestinal epithelial barrier (41, 42). In this study performed under non-challenging conditions, oral supplementation with YCW1, 2 and 3 elevated goblet cell density (GCD) and mucin acidic chemotypes (**Figures 2A, B**). This agrees with other studies in terrestrial and aquatic farmed species (17, 43–47). Interestingly, acidic mucins have been shown to help scavenge free hydroxyl radicals and increase mucus viscosity (48, 49). Given the apparent elevations in GCD in fish fed the YCW fractions with higher levels of mannoproteins, future studies should focus towards investigating the effects of mannoproteins on GC composition particularly GC chemotypes and mucin composition as this is seldom reported across FFA studies.

Further, the abundance of IEL in the intestinal barrier was measured. In humans, Intestinal IELs are a frontline heterogeneous subpopulation of T-cells and the TCRγδ+ T cells represent about 10% of the lymphocyte population in the small intestine. Their primary function is to maintain intestinal homeostasis and epithelial barrier function by providing immunosurveillance and effector immune functions against pathogen translocation through innate-like mechanisms or as antigen-specific memory T-cells (50). Accordingly in teleost fish, the posterior intestine is an area of high pinocytosis and heightened antigen uptake and surveillance by IELs that are thought to be mainly CD8α^+^ TCRγδ^+^ T cells (51–53). Although in the current study there was no direct staining of the IEL population, staining the intestinal tissue with H&E revealed significantly elevated presence of IELs in the PI of fish fed YCW1, 2 and 3. IELs hyperplasia upon whole-YCW supplementation was previously documented in broilers (54), as well as in the European seabass (55). In contrast to YCW4, YCW1, 2 and 3 had a high mannan content and presented a high level of α-mannans of varying chain-length. The mannan and more specifically α-mannans content and bioactivity would therefore appear important features of YCWs to promote the expansion of intestinal goblet cells (GC), particularly of the acidic chemotype associated with increased mucus viscosity and buffering capacity, and of intestinal IELs in the submucosa. The mechanisms at play in such YCW responses are not fully elucidated. Yeast-derived MOS are ligands to the endocytic mannose-receptor (MR) primarily expressed on macrophages and dendritic cells (56, 57). MR ligation has been associated with in an array of mechanisms including phagocytosis, antigen processing, cell migration as well as intestinal homeostasis and the resolution of inflammatory processes (58). Besides mucin secretion, GCs form goblet cell-associated antigen passages (GAPs) able to deliver luminal antigens to antigen presenting cells in the submucosa for processing and presentation to IELs and other adaptive immune cells (41). In summary, YCW4 that contained exposed β-glucans had little apparent effect on the intestinal barrier responses unlike the mannan-rich YCW1, 2 and 3 fractions which promoted GC and IEL abundance. Such fortification of the intestinal barrier and immune competence can present an effective strategy to reduce host-adhesion and invasion by potential pathogens and suggest enhanced antigen sensing capacity (58). However, the histological appraisal of the intestinal mucosa only provides a limited view on the potentially specific immune functionalities of contrasted YCWs.

To verify the ability of the four different *S. cerevisiae* YCW to elicit a different intestinal immune response, different classes of innate immune PRRs were investigated including scavenger receptors (SRs), microbe sensing toll-like receptors (TLRs) and downstream signal transduction markers. These PRRs have previously been implicated in the detection of microbial associated molecular patterns (MAMPs) from the *S. cerevisiae* yeast (59–62). The four YCW fractions assessed in this study displayed different affinity to the different PRRs assessed based on their gene expression responses. YCW4 and YCW1 both displayed significant elevations in expression of *tlr2, tlr4* and *marco* despite marked differences in cell wall composition and structure. On the other hand, YCW2 and 3 had similar mannans and β-glucans composition; but elicited distinctive PRR-responses reflecting their contrasted YCW architecture. It therefore appears that the cell wall composition does not adequately predict PRRs recognition such that *S. cerevisae* YCWs containing similar levels of mannans, and glucans could lead to distinctive downstream signalling and immune responses.

Different studies have shown that the structure of β-glucans will influence the recognition and subsequent immunomodulatory effects of this polysaccharide, where large molecular weight and particulate β-glucans are mainly recognised by TLR2 as reviewed by Brown and Gordon (63). Our results appear to be in agreement as YCW2 displayed the largest β-1,3-glucan chain lengths and elicited the highest *tlr2* up-regulation followed by YCW3 and 4. Mannan chain lengths may also have had a significant effect on PRR recognition of YCW fractions 2, 3 and 4. Indeed, Nigou and colleagues (64) reported that fungal extracts presenting longer mannan chain lengths had a significantly higher affinity to TLR2 which modified downstream signalling. Accordingly in this study, the longer mannan chain lengths of YCW2, 3 and 4 compared to YCW1 may also have contributed to the upregulating of *tlr2* expression. Besides TLR2, there are recognised synergistic relationships between dectin-1, a major β-glucan receptor, and TLR4 for recognition of β-glucan and mannan ligands (65–67). Interestingly, YCW fractions 1 and 4 displayed a shorter mannan length (**Figure 1D**) that could confer better accessibility to the β-1,3-glucan located beneath the outer mannan layer of the yeast cell wall. This may have influenced the potential binding to TLR4 as *tlr4* expression level was significantly elevated in fish fed YCW1 and 4 compared to the control (**Figure 3B**).

The study also measured the expression levels of the class A scavenger receptor *marco* that is mainly expressed on macrophages and plays a major role in the antibacterial host defences as confirmed in fish (68). Indeed, using MARCO knockout transgenic mice lines, Bowdish and colleagues (69) reported that MARCO was an important receptor required for TLR signalling during *Mycobacterium tuberculosis* infection. Notably, macrophages from MARCO defective mice were unable to secrete pro-inflammatory cytokines in the presence of *M. tuberculosis*. Besides, MARCO is involved in the direct recognition of major constituents of fungal cell walls such as yeasts and eradication of fungal pathogens (70). In this study, the expression level of *marco* was significantly elevated compared to the control in fish exposed to YCW fractions 1, 3 and 4 but not to YCW fraction 2. Interestingly, in relation to YCW structure the contour lengths for β-1,3-glucans were shorter by ~10% in YCW fractions 1, 3 and 4 compared to YCW fraction 2 (**Figure 1D**). Accordingly, the shorter β-glucan chain lengths may require cooperation of PRRs including MARCO to “tether” the yeast ligands to macrophages and activate either TLR2 or TLR4.

Following recognition of YCWs MAMPs, the subsequent signalling indicated activation of immune receptors as the expression levels for *myd88* were significantly elevated in all fish exposed to the YCW fractions (**Figure 3D**). In contrast the expression of *traf6*, which mediates IL-1 signalling, was significantly elevated in fish exposed to YCW fractions 3 and 4 only. Notably, TRAF6 has been implicated to play a protective role in epithelial barrier homeostasis and innate protective response in the intestine (71). Likewise, the expression of *tollip 1* gene, an adaptor-protein associating directly with TLR2 and TLR4 and playing an inhibitory role in TLR-mediated cell activation (72), was significantly elevated with YCW fractions 1, 3 and 4 only (**Figure 3E**). It would therefore appear that although all YCW tested induced the expression of markers for TLR-MyD88 signalling, some but not all fractions show the potential to further regulate TLR-mediated signalling *via* induction of TOLLIP1 gene; again, pointing to an impact of the yeast cell wall architecture on its immunogenicity.

It must be emphasised that yeast β-glucans and mannans are known to interact with an array of PRRs including Dectin 1 and 2, DC-SIGN, TLR2, TLR4, and TLR6 located on various immune cells such as monocytes, macrophages, neutrophils and T-regs (73, 74). Future studies should expand the PRRs target to profile MAMP’s-PRR interactions across yeast cell wall fractions of distinctive molecular structure. This was not performed in this study, which focused on gene expression markers for TLR-MyD88 signalling through to transcription factors, cytokine and intestinal tissue responses in order to document the distinctive immune properties of *S. cerevisae* derived yeast cell wall fractions from detection to tissue response.

The main cytokines families in teleosts are the interleukins (ILs), interferons (IFN), tumor necrosis factors (TNF), and transforming growth factors (TGF) produced by several innate as well as adaptive immune cells. Cytokines orchestrate innate immunity and further characterise the adaptive immune response hence have a pivotal role in the clearance of infectious agents (75, 76). From this perspective, the study characterised the gut immune response using molecular markers for characterisation of both effective innate and T-cell mediated immunity.

**Figure 6:**
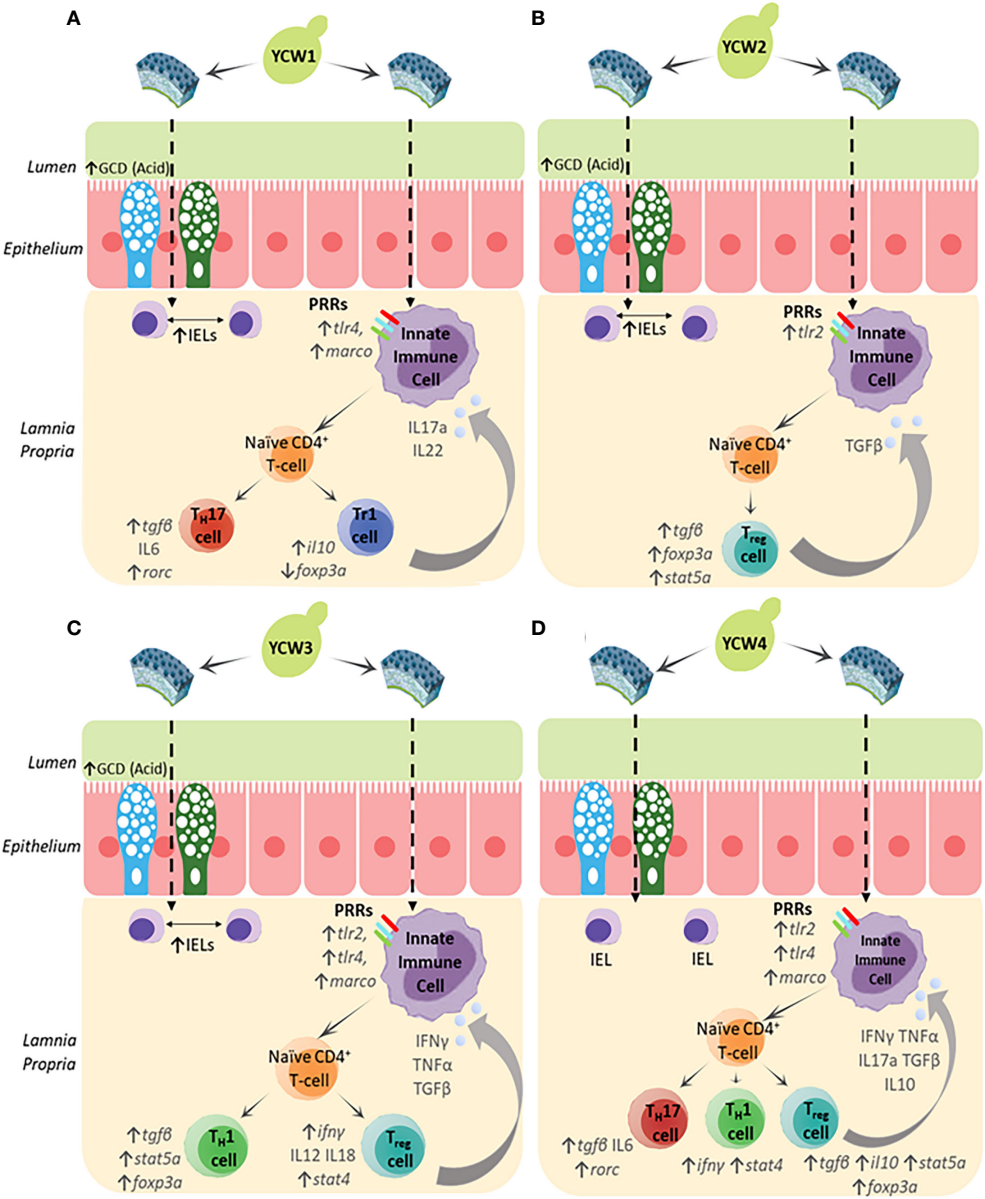
The biochemical and molecular characterisation of the YCW fractions **(A)** YCW 1, **(B)** YCW 2, **(C)** YCW 3 and **(D)** YCW 4, demonstrate elevations in specific biomarkers for fortification of intestinal barrier integrity (↑ GCD (Acidomucins), ↑IELs) except for YCW fraction 4. Apparent differences in immune competence markers are proposed to have been driven by differences in cell wall mannan and β-glucan content as well as structure determining potential recognition by different PRRs presented on innate immune cells such as macrophages (Mϕ), dendritic cells and neutrophils present in the underlying intestinal tissue. Downstream signalling suggests potential mobilisation of naïve T-helper cells to effector subsets and drive specific cytokine milieus to control innate immunity.

Herein the results showed that different YCW fractions elicited specific gene expression profiles for transcription factors (TFs) and effector cytokines that reflected mobilisation of effector T-helper cell subsets for Th17, Th1, Tr1 and Th3 (**Figure 6**). Oral administration of YCW1 and 4 elicited the upregulation of a cluster of genes suggestive of polarisation of naïve T-cells to Th17-like cells including *tlr4*, *myd88*, *stat4*, *rorc* and high expression for *il17a* compared to all other experimental groups. RAR-related orphan receptor gamma (RORγ) is a protein that in humans is encoded by the RORC (RAR-related orphan receptor C) gene and the induction of transcription factor RORγt is a key part of the transcriptional programming required for Th17 cell differentiation. The effector cytokines IL-17 and granulocyte macrophage colony-stimulating factor (GM-CSF) are integral to the recruitment of neutrophils to the site of inflammation, promoting inflammatory processes (77, 78). Th17 cell-mediated immunity (CMI) has been shown to be effective at removing extracellular fungal and bacterial pathogens such as *Klebsiella pneumonia*, *Citrobacter rodentium* and *Candida albicans* (79). Here we show evidence of polarisation of naive T-cells to Th17-like cells in the form of significant upregulation in the expression of *rorc* and *il17a* in experimental groups YCW1 and 4 compared to the control group. Interestingly, this is in line with our present findings that YCW1 and 4 elicited elevations in the expression of *tlr4* which has been shown to directly regulate Th17 differentiation (80). However, zebrafish were recently found to present ILC-like cells involved in the mucosal immune response and homeostasis of mammals (81). Like Th17 T-cells, ILC3s require the induction of RORC for activation and produce effector cytokines IL-17a, IL-22 and GM-CSF. Accordingly, PRR recognition of yeast ligands *via* a combination of C-type lectin receptors and, or TLR4 could influence cell signalling and effector cytokines for polarisation of naïve T-cells to Th17-like cells or activation of ILC3s, as shown in studies with human cell lines and mice (82–84). The short mannans chain-length measured in both YCW1 and 4 (**Figure 1D**) may have constituted an important trait to elicit such downstream immune functions.

The pro-inflammatory Th1 cell-mediated responses are orchestrated through the release of signature Th1 cytokines, IFN-γ and TNF-α (85, 86). Indeed, these cytokines play a pivotal role in the induction of classically activated (M1) macrophages which assist in the clearance of both fungal and intracellular pathogens (87). Furthermore, transcription factors STAT4 and T-bet are important mediators in the differentiation of naïve T-cells to Th1 subsets and STAT4 is preferentially expressed in Th1 cells (88, 89). Herein compared to the control group, oral administration of YCW fractions 3 and 4 elevated *tlr2* and *myd88* (**Figures 3A, D**), *stat4* and *rorc* (**Figures 6A, B**), *tnfα* and *ifnγ* (**Figure 5A**) expression level together suggesting the potential polarisation of naïve T-cells to Th1-cells *via* TLR2-Myd88 signalling. In contrast, YCW 4 displayed shorter mannan chains but longer β-1,3-glucan chains conceivably allowing for exposure of β-glucans. Indeed, as aforementioned, it was apparent that fish fed the YCW fraction 4 displayed significant elevations in all PRR genes showing the potential for a strong affinity to MARCO-Syk, TLR2 and TLR4-MyD88 signalling. This in part could explain why the expression profile for YCW4 suggest mobilisation of effector cytokines and polarisation of naïve CD4 T-cells to Th1/Th17 subsets.

Regulation of the immune response is important in protecting the host from infection-associated immunopathology, autoimmune diseases and allergy. At the heart of these immunological events are regulatory T-cells (Treg) that are key to orchestrating the immune response and tissue repair. Treg cells are abundant in non-lymphoid tissues particularly at mucosal surfaces where constant exposure to inflammatory triggers must be tightly regulated to ensure homeostasis at a steady state or be ready to engage in potent immune responses when required. In this regard, the study targeted key effector cytokines (IL-10 and TGF-β) and master transcription factors (FoxP3 and stat5a) involved in the polarisation of naïve T-cells to Treg cells (90). Results revealed distinct patterns for stimulation of secondary inducible T regulatory cells (iTregs) analogous to T regulatory type 1 (Tr1) cells and FOXP3^+^ iTreg cells.

In particular, oral exposure of YCW fraction 1 significantly elevated the gene expression *il10* but not *foxp3a*, (**Figures 4A**, **6D**, respectively) reflecting a mobilisation of FOXP3^-^ Tr1 cells (91). Tr1 cells are typically located in the intestinal mucosa and their key role is to maintain peripheral tolerance and suppress tissue inflammation to self and non-self antigens. Interestingly, human Tr1 cells have been reported to secrete IL-22 an IL-10 family member cytokines that act on intestinal epithelial cells to promote intestinal cell barrier integrity by driving mucin production and differentiation (92). This agrees with our histological findings of a higher goblet cell density and altered chemotypes in fish fed the YCW1 fraction (**Figures 2A, C**). In contrast and compared to the control, fish fed YCW2, 3, and 4 fractions significantly elevated the expression levels of *tgfβ*, *stat5a* and *foxp3a* (**Figures 5A**, **4C, D**, respectively). These profiles may reflect a polarisation of naïve T-cells to FOXP3^+^ iTreg cells that are essential in tolerance to self and non-self antigens (93). To summarise, the markers assessed suggested contrasted effects on iTreg subsets in response to the presentation of different yeast cell wall polysaccharides. Recently, a preparation of mannan/β-1,6-glucan was found to facilitate the induction of Treg from naïve T-cells by a Dectin1-Cox2 signalling pathway (94). This confirms the potential of yeast-fractions as T-reg immunity modifiers as well as the interest of assessing the affinity of YCW fractions to augment specific PRR signalling pathways in future studies.

## 5 Conclusion

The study documented marked intra-species variability in the molecular properties of *S. cerevisae* yeast cell wall fractions from discrete sources which were associated with contrasted mucosal immune responses upon oral administration in a vertebrate animal model. The study identifies α-mannan content as a potent driver of GCD and IEL hyperplasia, suggestive of fortifying intestinal barrier integrity and immune competence. Further the structural molecular differences of the YCW polysaccharides, in terms of α-mannans and β-1,3-glucans chain-length, are shown to modify the expression pattern of PRR responses.

The resulting downstream transcription factors and cytokine responses suggest the preferential mobilisation of distinct effector T-helper cell subsets for Th17, Th1, Tr1 and Foxp3^+^-Treg (**Figure 6**), indicating a particular potential for each YCW fraction against infectious agents and, or non-infectious pathologies (**Table 3**). Although this study under no challenge condition suggests priming responses on adaptive immune T cells but cannot, yet, be discriminated from the functionality of other cells, such as innate lymphoid cells (ILCs) which are found in the gut mucosa. Further investigation with a time-course of immune response to pathogenic/antigen challenge can be interesting for future investigation of the adaptive immune response. Accordingly, beyond the mannans and β-1,3/β-1,6-glucans content of *S. cerevisae* YCW fraction, the study confirms the importance of considering the molecular structural characteristics of the YCW to apprehend their specific immune properties and ultimately elicit targeted immune-functionalities. This comparative study offers new perspectives in the development of specific *S. cerevisae* fractions towards targeted application in precision animal nutrition.

**Table 3:** Specific functional responses of each YCW fraction related to their effectiveness at clearing different pathogen types and maintenance of immune homeostasis.

Functional responses	YCW1	YCW2	YCW3	YCW4
Pro-inflammatory	Extracellular anti-bacterial/fungal CMI – Th17 response	No response	Intracellular anti-viral/bacterial CMI –Th1 response	Anti-viral/bacterial/fungal CMI – Th1/17 response
Anti-inflammatory	Non-specific tolerance/tissue repair CMI – Tr1 (Foxp3^-^)	Tissue repair and immune homeostasis CMI – iTreg (Foxp3^+^)	Tissue repair and immune homeostasis CMI – iTreg (Foxp3^+^)	Tissue repair and immune homestasis CMI – iTreg (Foxp3^+^)

## Data availability statement

The original contributions presented in the study are included in the article/**Supplementary Material**. Further inquiries can be directed to the corresponding authors.

## Ethics statement

The animal study was reviewed and approved by University of Plymouth animal ethical review board.

## Author contributions

MR, DM, EL, MC, and AF contributed to conception and design of the study. MR, MS, and EL organised the data for submission. MR and MS performed the statistical analysis. MR, MS, EL, and EA wrote the first draft of the manuscript. MR, MS, and EL wrote sections of the manuscript. All authors contributed to the article and approved the submitted version.

## References

[1] Donaldson DS Else KJ Mabbott NA. The gut-associated lymphoid tissues in the small intestine, not the large intestine, play a major role in oral prion disease pathogenesis. J Virol (2015) 89(18):9532–47. doi: 10.1128/JVI.01544-15 PMC4542385 26157121

[2] Salinas I. The mucosal immune system of teleost fish. Biology (2015) 4(3):525–39. doi: 10.3390/biology4030525 PMC4588148 26274978

[3] Mörbe UM Jørgensen PB Fenton TM von Burg N Riis LB Spencer J. Human gut-associated lymphoid tissues (GALT); diversity, structure, and function. Mucosal Immunol (2021) 14(4):793–802. doi: 10.1038/s41385-021-00389-4 33753873

[4] Sichetti M De Marco S Pagiotti R Traina G Pietrella D. Anti-inflammatory effect of multistrain probiotic formulation (L. rhamnosus, b. lactis, and b. longum). Nutrition (2018) 53:95–102. doi: 10.1016/j.nut.2018.02.005 29674267

[5] McCracken VJ Lorenz RG. The gastrointestinal ecosystem: a precarious alliance among epithelium, immunity and microbiota: microreview. Cell Microbiol (2001) 3(1):1–1. doi: 10.1046/j.1462-5822.2001.00090.x 11207615

[6] Lickwar CR Camp JG Weiser M Cocchiaro JL Kingsley DM Furey TS. Genomic dissection of conserved transcriptional regulation in intestinal epithelial cells. PloS Biol (2017) 15(8):e2002054. doi: 10.1371/journal.pbio.2002054 28850571 PMC5574553

[7] Attaya A Wang T Zou J Herath T Adams A Secombes CJ. Gene expression analysis of isolated salmonid GALT leucocytes in response to PAMPs and recombinant cytokines. Fish shellfish Immunol (2018) 80:426–36. doi: 10.1016/j.fsi.2018.06.022 29906623

[8] Gomes MC Mostowy S. The case for modeling human infection in zebrafish. Trends Microbiol (2020) 28(1):10–8. doi: 10.1016/j.tim.2019.08.005 31604611

[9] Scapigliati G Fausto AM Picchietti S. Fish lymphocytes: an evolutionary equivalent of mammalian innate-like lymphocytes? Front Immunol (2018) 9:971. doi: 10.3389/fimmu.2018.00971 29867952 PMC5949566

[10] Flajnik MF. A cold-blooded view of adaptive immunity. Nat Rev Immunol (2018) 18(7):438–53. doi: 10.1038/s41577-018-0003-9 PMC6084782 29556016

[11] Buddington RK Krogdahl A Bakke-McKellep AM. The intestines of carnivorous fish: structure and functions and the relations with diet. Acta Physiologica Scandinavica Supplementum (1997) 638:67–80. 9421581

[12] Swan CM Lindstrom NM Cain KD. Identification of a localized mucosal immune response in rainbow trout, *Oncorhynchus mykiss* (Walbaum), following immunization with a protein-hapten antigen. J Fish Dis (2008) 31(5):383–93. doi: 10.1111/j.1365-2761.2008.00918.x 18400055

[13] Sunyer JO. Fishing for mammalian paradigms in the teleost immune system. Nat Immunol (2013) 14(4):320–6. doi: 10.1038/ni.2549 PMC4203445 23507645

[14] Howe K Clark MD Torroja CF Torrance J Berthelot C Muffato M. The zebrafish reference genome sequence and its relationship to the human genome. Nature (2013) 496(7446):498–503. doi: 10.1038/nature12111 23594743 PMC3703927

[15] van der Vaart M Spaink HP Meijer AH. Pathogen recognition and activation of the innate immune response in zebrafish. Adv Hematol (2012) 2012. doi: 10.1155/2012/159807 PMC3395205 22811714

[16] Wan F Hu CB Ma JX Gao K Xiang LX Shao JZ. Characterization of γδ T cells from zebrafish provides insights into their important role in adaptive humoral immunity. Front Immunol (2017) 7:675. doi: 10.3389/fimmu.2016.00675 28119690 PMC5220103

[17] Torrecillas S Montero D Izquierdo M. Improved health and growth of fish fed mannan oligosaccharides: potential mode of action. Fish Shellfish Immunol (2014) 36(2):525–44. doi: 10.1016/j.fsi.2013.12.029 24412165

[18] Zentek J Marquart B Pietrzak T. Intestinal effects of mannanoligosaccharides, transgalactooligosaccharides, lactose and lactulose in dogs. J Nutr (2002) 132(6):1682S–4S. doi: 10.1093/jn/132.6.1682S 12042492

[19] Swanson KS Grieshop CM Flickinger EA Bauer LL Healy HP Dawson KA. Supplemental fructooligosaccharides and mannanoligosaccharides influence immune function, ileal and total tract nutrient digestibilities, microbial populations and concentrations of protein catabolites in the large bowel of dogs. J Nutr (2002) 132(5):980–9. doi: 10.1093/jn/132.5.980 11983825

[20] Upadhaya SD Bravo de Laguna F Bertaud B Kim IH. Multi-strain yeast fraction product supplementation can alleviate weaning stress and improve performance and health of piglets raised under low sanitary conditions. J Sci Food Agriculture (2019) 99(13):6076–83. doi: 10.1002/jsfa.9885 31233219

[21] Zhou H Yu B He J Mao X Zheng P Yu J. The optimal combination of dietary starch, non-starch polysaccharides, and mannan-oligosaccharide increases the growth performance and improves butyrate-producing bacteria of weaned pigs. Animals (2020) 10(10):1745. doi: 10.3390/ani10101745 32992960 PMC7600330

[22] Orlean P. Architecture and biosynthesis of the saccharomyces cerevisiae cell wall. Genetics (2012) 192(3):775–818. doi: 10.1534/genetics.112.144485 23135325 PMC3522159

[23] Fleet GH. Cell walls. In: Rose AH Harrison JS, editors. The yeasts, 2, vol. 4 . New York: Academic Press (1991). p. 199–277.

[24] Brown GD Gordon S. Fungal β-glucans and mammalian immunity. Immunity (2003) 19(3):311–5. doi: 10.1016/S1074-7613(03)00233-4 14499107

[25] Van der Werf MJ Zollstrasse W. MOS products: not every yeast cell wall is created equal. In: (Hamburg, Germany:Ohly Application Note (2019). Available at: www.ohly.com/en/feed-health.

[26] National Research Council. Nutritional requirements of fish. Washington DC: national academy press (2012).

[27] AOAC. Method 2007-04. Association of official analytical chemists. (Washington, DC: Springer) (2007).

[28] Francois JM. A simple method for quantitative determination of polysaccharides in fungal cell walls. Nat Protoc (2006) 1(6):2995–3000. doi: 10.1038/nprot.2006.457 17406560

[29] Formosa C Pillet F Schiavone M Duval RE Ressier L Dague E. Generation of living cell arrays for atomic force microscopy studies. Nat Protoc (2015) 10(1):199–204. doi: 10.1038/nprot.2015.004 25551664

[30] Schiavone M Sieczkowski N Castex M Trevisiol E Dague E François JM. AFM dendritips functionalized with molecular probes specific to cell wall polysaccharides as a tool to investigate cell surface structure and organization. Cell Surface (2019) 5:100027. doi: 10.1016/j.tcsw.2019.100027 32743143 PMC7389267

[31] Bustamante C Marko JF Siggia ED Smith S. Entropic elasticity of lambda-phage DNA. Science (1994) 265(5178):1599–600. doi: 10.1126/science.8079175 8079175

[32] Rawling M Leclercq E Foey A Castex M Merrifield D. A novel dietary multi-strain yeast fraction modulates intestinal toll-like-receptor signalling and mucosal responses of rainbow trout (*Oncorhynchus mykiss*). PloS One (2021) 16(1):e0245021. doi: 10.1371/journal.pone.0245021 33434201 PMC7802930

[33] Bustin SA Benes V Garson JA Hellemans J Huggett J Kubista M. The MIQE guidelines: minimum information for publication of quantitative real-time PCR experiments. Clin Chem (2009) 55(4):611–22. doi: 10.1373/clinchem.2008.112797 19246619

[34] R Core Team. R: a language and environment for statistical computing. Vienna, Austria: R Foundation for Statistical Computing (2022). Available at: https://www.R-project.org/.

[35] Ohmel JR. Precision intervals for estimates of the difference in success rates for binary random variables based on the permutation principle. Biometrical J (1996) 38:977–93. doi: 10.1002/bimj.4710380810

[36] Oksanen J Blanchet FG Kindt R Legendre P Minchin PR O’hara RB. Package ‘vegan’. Community Ecol package version (2013) 2(9):1–295.

[37] Netea MG Latz E Mills KH O'neill LA. Innate immune memory: a paradigm shift in understanding host defense. Nat Immunol (2015) 16(7):675–9. doi: 10.1038/ni.3178 26086132

[38] Posadas GA Broadway PR Thornton JA Carroll JA Lawrence A Corley JR. Yeast pro-and paraprobiotics have the capability to bind pathogenic bacteria associated with animal disease. Trans Anim Sci (2017) 1(1):60–8. doi: 10.2527/tas2016.0007 PMC7011128 32064460

[39] Akramienė D Kondrotas A Didžiapetrienė J Kėvelaitis E. Effects of ß-glucans on the immune system. Medicina (2007) 43(8):597. doi: 10.3390/medicina43080076 17895634

[40] Brugman S. The zebrafish as a model to study intestinal inflammation. Dev Comp Immunol (2016) 64:82–92. doi: 10.1016/j.dci.2016.02.020 26902932

[41] McDole JR Wheeler LW McDonald KG Wang B Konjufca V Knoop KA. Goblet cells deliver luminal antigen to CD103+ dendritic cells in the small intestine. Nature (2012) 483(7389):345–9. doi: 10.1038/nature10863 PMC3313460 22422267

[42] Knoop KA McDonald KG McCrate S McDole JR Newberry RD. Microbial sensing by goblet cells controls immune surveillance of luminal antigens in the colon. Mucosal Immunol (2015) 8(1):198–210. doi: 10.1038/mi.2014.58 25005358 PMC4268115

[43] Rodriguez-Estrada U Satoh S Haga Y Fushimi H Sweetman J. Effects of single and combined supplementation of enterococcus faecalis, mannan oligosaccharide and polyhydroxybutyrate acid on growth performance and immune response of rainbow trout oncorhynchus mykiss. Aquaculture Sci (2009) 57(4):609–17.

[44] Torrecillas S Makol A Caballero MJ Montero D Ginés R Sweetman J. Improved feed utilization, intestinal mucus production and immune parameters in sea bass (Dicentrarchus labrax) fed mannan oligosaccharides (MOS). Aquaculture Nutr (2011) 17(2):223–33. doi: 10.1111/j.1365-2095.2009.00730.x

[45] Lea H Spring P Taylor-Pickard J Burton E. A natural carbohydrate fraction actigen™ from saccharomyces cerevisiae cell wall: effects on goblet cells, gut morphology and performance of broiler chickens. J Appl Anim Nutr (2012) 1:e9. doi: 10.1017/jan.2013.6

[46] Song M Fan Y Su H Ye J Liu F Zhu X. Effects of actigen, a second-generation mannan rich fraction, in antibiotics-free diets on growth performance, intestinal barrier functions and inflammation in weaned piglets. Livestock Sci (2019) 229:4–12. doi: 10.1016/j.livsci.2019.09.006

[47] Leclercq E Pontefract N Rawling M Valdenegro V Aasum E Andujar LV. Dietary supplementation with a specific mannan-rich yeast parietal fraction enhances the gut and skin mucosal barriers of Atlantic salmon (Salmo salar) and reduces its susceptibility to sea lice (Lepeophtheirus salmonis). Aquaculture (2020) 529:735701. doi: 10.1016/j.aquaculture.2020.735701

[48] Croix JA Carbonero F Nava GM Russell M Greenberg E Gaskins HR. On the relationship between sialomucin and sulfomucin expression and hydrogenotrophic microbes in the human colonic mucosa. PloS One (2011) 6(9):e24447. doi: 10.1371/journal.pone.0024447 21931721 PMC3170330

[49] Ogasawara Y Namai T Yoshino F Ishii K. Sialic acid is an essential moiety of mucin as a hydroxyl radical scavenger. FEBS Lett (2007) 581(13):2473–7. doi: 10.1016/j.febslet.2007.04.062 17485090

[50] Sheridan BS Lefrançois L. Intraepithelial lymphocytes: to serve and protect. Curr Gastroenterol Rep (2010) 12:513–21. doi: 10.1007/s11894-010-0148-6 PMC3224371 20890736

[51] Rombout JH Yang G Kiron V. Adaptive immune responses at mucosal surfaces of teleost fish. Fish Shellfish Immunol (2014) 40(2):634–43. doi: 10.1016/j.fsi.2014.08.020 25150451

[52] Rombout JH Abelli L Picchietti S Scapigliati G Kiron V. Teleost intestinal immunology. Fish Shellfish Immunol (2011) 31(5):616–26. doi: 10.1016/j.fsi.2010.09.001 20832474

[53] Bjørgen H Li Y Kortner TM Krogdahl Å Koppang EO. Anatomy, immunology, digestive physiology and microbiota of the salmonid intestine: knowns and unknowns under the impact of an expanding industrialized production. Fish Shellfish Immunol (2020) 107:172–86. doi: 10.1016/j.fsi.2020.09.032 32979510

[54] Gao J Zhang HJ Wu SG Yu SH Yoon I Moore D. Effect of saccharomyces cerevisiae fermentation product on immune functions of broilers challenged with *Eimeria tenella* . Poultry Sci (2009) 88(10):2141–51. doi: 10.3382/ps.2009-00151 19762868

[55] Sheng KC Pouniotis DS Wright MD Tang CK Lazoura E Pietersz GA. Mannan derivatives induce phenotypic and functional maturation of mouse dendritic cells. Immunology (2006) 118(3):372–83. doi: 10.1111/j.1365-2567.2006.02384.x PMC1782308 16827898

[56] Ringø E Olsen RE Gifstad TØ Dalmo RA Amlund H Hemre GI. Prebiotics in aquaculture: a review. Aquaculture Nutr (2010) 16(2):117–36. doi: 10.1111/j.1365-2095.2009.00731.x

[57] Gazi U Martinez-Pomares L. Influence of the mannose receptor in host immune responses. Immunobiology (2009) 214(7):554–61. doi: 10.1016/j.imbio.2008.11.004 19162368

[58] van der Marel M Adamek M Gonzalez SF Frost P Rombout JH Wiegertjes GF. Molecular cloning and expression of two β-defensin and two mucin genes in common carp (*Cyprinus carpio* l.) and their up-regulation after β-glucan feeding. Fish Shellfish Immunol (2012) 32(3):494–501. doi: 10.1016/j.fsi.2011.12.008 22227003

[59] Netea MG van der Graaf CA Vonk AG Verschueren I van der Meer JW Kullberg BJ. The role of toll-like receptor (TLR) 2 and TLR4 in the host defense against disseminated candidiasis. J Infect Dis (2002) 185(10):1483–9. doi: 10.1086/340511 11992285

[60] Tada H Nemoto E Shimauchi H Watanabe T Mikami T Matsumoto T. Saccharomyces cerevisiae-and candida albicans-derived mannan induced production of tumor necrosis factor alpha by human monocytes in a CD14-and toll-like receptor 4-dependent manner. Microbiol Immunol (2002) 46(7):503–12. doi: 10.1111/j.1348-0421.2002.tb02727.x 12222939

[61] Brown GD Gordon S. Immune recognition of fungal β-glucans. Cell Microbiol (2005) 7(4):471–9. doi: 10.1111/j.1462-5822.2005.00505.x 15760447

[62] Taylor PR Brown GD Herre J Williams DL Willment JA Gordon S. The role of SIGNR1 and the β-glucan receptor (dectin-1) in the nonopsonic recognition of yeast by specific macrophages. J Immunol (2004) 172(2):1157–62. doi: 10.4049/jimmunol.172.2.1157 14707091

[63] Józefowski S Yang Z Marcinkiewicz J Kobzik L. Scavenger receptors and β-glucan receptors participate in the recognition of yeasts by murine macrophages. Inflamm Res (2012) 61:113–26. doi: 10.1007/s00011-011-0395-5 PMC3265724 22116297

[64] Brown GD Gordon S. Fungal beta-glucans and mammalian immunity. Immunity (2003) 19(3):311–5. doi: 10.1016/S1074-7613(03)00233-4 14499107

[65] Nigou J Vasselon T Ray A Constant P Gilleron M Besra GS. Mannan chain length controls lipoglycans signaling *via* and binding to TLR2. J Immunol (2008) 180(10):6696–702. doi: 10.4049/jimmunol.180.10.6696 18453589

[66] Sahasrabudhe NM Dokter-Fokkens J de Vos P. Particulate β-glucans synergistically activate TLR4 and dectin-1 in human dendritic cells. Mol Nutr Food Res (2016) 60(11):2514–22. doi: 10.1002/mnfr.201600356 27358258

[67] Zhang Y Liu X Zhao J Wang J Song Q Zhao C. The phagocytic receptors of β-glucan. Int J Biol Macromolecules (2022) 205:430–41. doi: 10.1016/j.ijbiomac.2022.02.111 35202631

[68] Benard EL Roobol SJ Spaink HP Meijer AH. Phagocytosis of mycobacteria by zebrafish macrophages is dependent on the scavenger receptor Marco, a key control factor of pro-inflammatory signalling. Dev Comp Immunol (2014) 47(2):223–33. doi: 10.1016/j.dci.2014.07.022 25086293

[69] Bowdish DM Sakamoto K Kim MJ Kroos M Mukhopadhyay S Leifer CA. MARCO, TLR2, and CD14 are required for macrophage cytokine responses to mycobacterial trehalose dimycolate and mycobacterium tuberculosis. PloS Pathogens (2009) 5(6):e1000474. doi: 10.1371/journal.ppat.1000474 19521507 PMC2688075

[70] Bin LH Nielson LD Liu X Mason RJ Shu HB. Identification of uteroglobin-related protein 1 and macrophage scavenger receptor with collagenous structure as a lung-specific ligand-receptor pair. J Immunol (2003) 171(2):924–30. doi: 10.4049/jimmunol.171.2.924 12847263

[71] Vlantis K Polykratis A Welz PS van Loo G Pasparakis M Wullaert A. TLR-independent anti-inflammatory function of intestinal epithelial TRAF6 signalling prevents DSS-induced colitis in mice. Gut (2016) 65(6):935–43. doi: 10.1136/gutjnl-2014-308323 PMC4893119 25761602

[72] Zhang G Ghosh S. Negative regulation of toll-like receptor-mediated signaling by tollip. J Biol Chem (2002) 277(9):7059–65. doi: 10.1074/jbc.M109537200 11751856

[73] Patin EC Thompson A Orr SJ. Pattern recognition receptors in fungal immunity. InSeminars Cell Dev Biol (2019) 89:24–33. doi: 10.1016/j.semcdb.2018.03.003 PMC6461132 29522806

[74] Stothers CL Burelbach KR Owen AM Patil NK McBride MA Bohannon JK. β-glucan induces distinct and protective innate immune memory in differentiated macrophages. J Immunol (2021) 207(11):2785–98. doi: 10.4049/jimmunol.2100107 PMC8612974 34740960

[75] Secombes CJ Zou J Bird S. Fish cytokines: discovery, activities and potential applications. Fish Defenses (2009) 1:1–36. doi: 10.1201/b10188-2

[76] Savan R Sakai M. Genomics of fish cytokines. Comp Biochem Physiol Part D: Genomics Proteomics (2006) 1(1):89–101. doi: 10.1016/j.cbd.2005.08.005 20483237

[77] Wilson AS Randall KL Pettitt JA Ellyard JI Blumenthal A Enders A. Neutrophil extracellular traps and their histones promote Th17 cell differentiation directly *via* TLR2. Nat Commun (2022) 13(1):528. doi: 10.1038/s41467-022-28172-4 35082281 PMC8792063

[78] Sandquist I Kolls J. Update on regulation and effector functions of Th17 cells. F1000Research (2018) 7. doi: 10.12688/f1000research.13020.1 PMC5820607 29527301

[79] Curtis MM Way SS. Interleukin-17 in host defence against bacterial, mycobacterial and fungal pathogens. Immunology (2009) 126(2):177–85. doi: 10.1111/j.1365-2567.2008.03017.x PMC2632692 19125888

[80] Park JH Jeong SY Choi AJ Kim SJ. Lipopolysaccharide directly stimulates Th17 differentiation *in vitro* modulating phosphorylation of RelB and NF-κB1. Immunol Lett (2015) 165(1):10–9. doi: 10.1016/j.imlet.2015.03.003 25794633

[81] Hernández PP Strzelecka PM Athanasiadis EI Hall D Robalo AF Collins CM. Single-cell transcriptional analysis reveals ILC-like cells in zebrafish. Sci Immunol (2018) 3(29):eaau5265. doi: 10.1126/sciimmunol.aau5265 30446505 PMC6258902

[82] Smeekens SP van de Veerdonk FL van der Meer JW Kullberg BJ Joosten LA Netea MG. The candida Th17 response is dependent on mannan-and β-glucan-induced prostaglandin E2. Int Immunol (2010) 22(11):889–95. doi: 10.1093/intimm/dxq442 21059767

[83] Netea MG Brown GD Kullberg BJ Gow NA. An integrated model of the recognition of candida albicans by the innate immune system. Nat Rev Microbiol (2008) 6(1):67–78. doi: 10.1038/nrmicro1815 18079743

[84] Verma A Wüthrich M Deepe G Klein B. Adaptive immunity to fungi. Cold Spring Harbor Perspect Med (2015) 5(3):a019612. doi: 10.1101/cshperspect.a019612 PMC4355251 25377140

[85] Elemam NM Hannawi S Maghazachi AA. Innate lymphoid cells (ILCs) as mediators of inflammation, release of cytokines and lytic molecules. Toxins (2017) 9(12):398. doi: 10.3390/toxins9120398 29232860 PMC5744118

[86] Wüthrich M Deepe GS Jr. Klein B. Adaptive immunity to fungi. Annu Rev Immunol (2012) 30:115–48. doi: 10.1146/annurev-immunol-020711-074958 PMC3584681 22224780

[87] Novak ML Koh TJ. Phenotypic transitions of macrophages orchestrate tissue repair. Am J pathology (2013) 183(5):1352–63. doi: 10.1016/j.ajpath.2013.06.034 PMC3969506 24091222

[88] Watford WT Hissong BD Bream JH Kanno Y Muul L O'Shea JJ. Signaling by IL-12 and IL-23 and the immunoregulatory roles of STAT4. Immunol Rev (2004) 202(1):139–56. doi: 10.1111/j.0105-2896.2004.00211.x 15546391

[89] Korman BD Kastner DL Gregersen PK Remmers EF. STAT4: genetics, mechanisms, and implications for autoimmunity. Curr Allergy Asthma Rep (2008) 8(5):398–403. doi: 10.1007/s11882-008-0077-8 18682104 PMC2562257

[90] Passerini L Allan SE Battaglia M Di Nunzio S Alstad AN Levings MK. STAT5-signaling cytokines regulate the expression of FOXP3 in CD4+ CD25+ regulatory T cells and CD4+ CD25– effector T cells. Int Immunol (2008) 20(3):421–31. doi: 10.1093/intimm/dxn002 18270368

[91] Song Y Wang N Chen L Fang L. Tr1 cells as a key regulator for maintaining immune homeostasis in transplantation. Front Immunol (2021) 12:671579. doi: 10.3389/fimmu.2021.671579 33981317 PMC8109434

[92] Cook L Stahl M Han X Nazli A MacDonald KN Wong MQ. Suppressive and gut-reparative functions of human type 1 T regulatory cells. Gastroenterology (2019) 157(6):1584–98. doi: 10.1053/j.gastro.2019.09.002 31513797

[93] Sakaguchi S Yamaguchi T Nomura T Ono M. Regulatory T cells and immune tolerance. Cell (2008) 133(5):775–87. doi: 10.1016/j.cell.2008.05.009 18510923

[94] Lee C Verma R Byun S Jeun EJ Kim GC Lee S. Structural specificities of cell surface β-glucan polysaccharides determine commensal yeast mediated immuno-modulatory activities. Nat Commun (2021) 12(1):3611. doi: 10.1038/s41467-021-23929-9 34127673 PMC8203763

