# Supplementary material for: Yeast cell wall extracts from Saccharomyces cerevisiae varying in structure and composition differentially shape the innate immunity and mucosal tissue responses of the intestine of zebrafish (Danio rerio)
Source: Front Immunol. 2023 May 25;14:1158390. doi: 10.3389/fimmu.2023.1158390 (PMC10248512; doi:10.3389/fimmu.2023.1158390)
Supplement: Supplementary file 1 [file Presentation_1.pptx]

## Slide 1
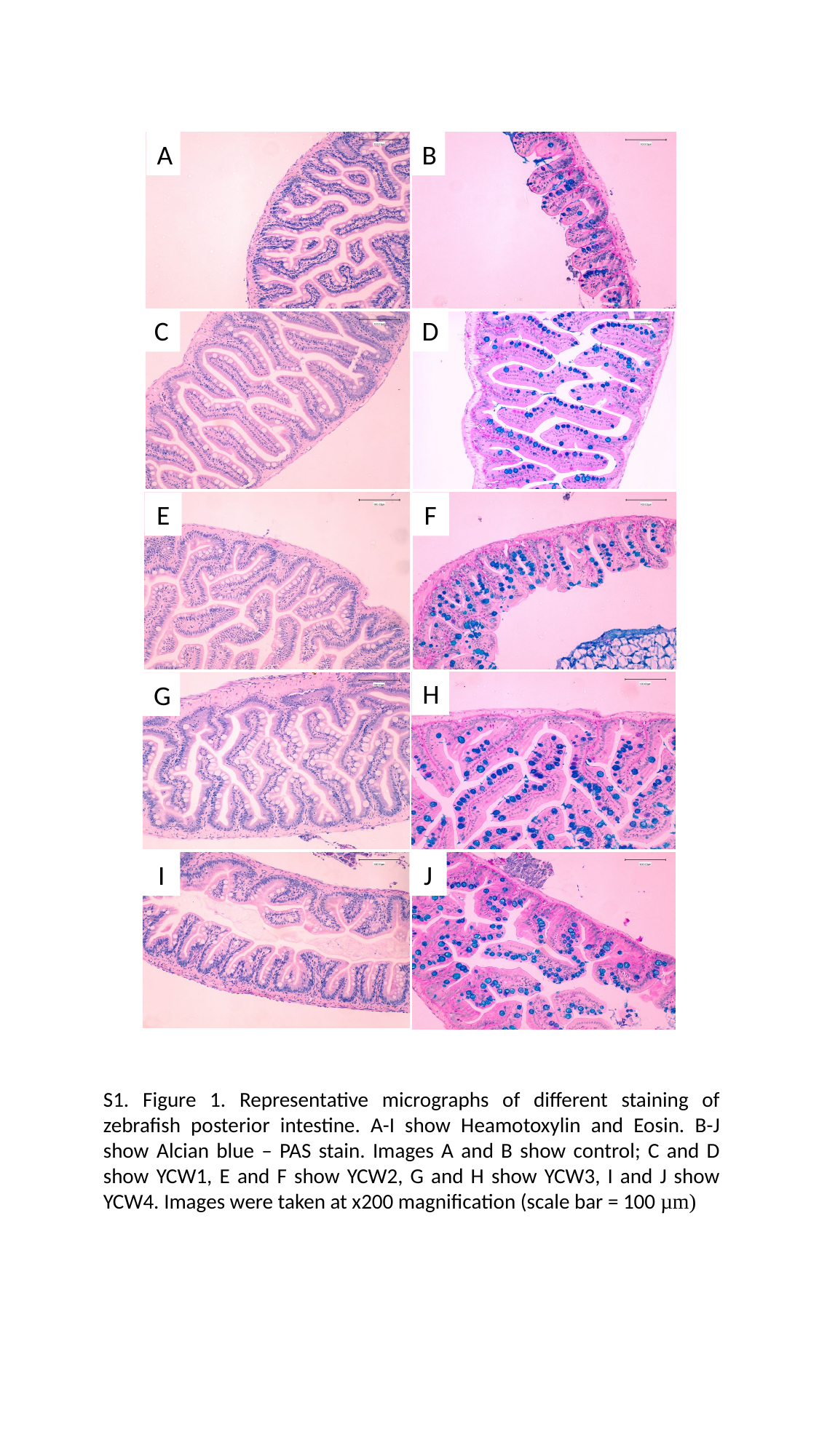

A
B
D
C
E
F
H
G
J
I
S1. Figure 1. Representative micrographs of different staining of zebrafish posterior intestine. A-I show Heamotoxylin and Eosin. B-J show Alcian blue – PAS stain. Images A and B show control; C and D show YCW1, E and F show YCW2, G and H show YCW3, I and J show YCW4. Images were taken at x200 magnification (scale bar = 100 µm)

## Slide 2
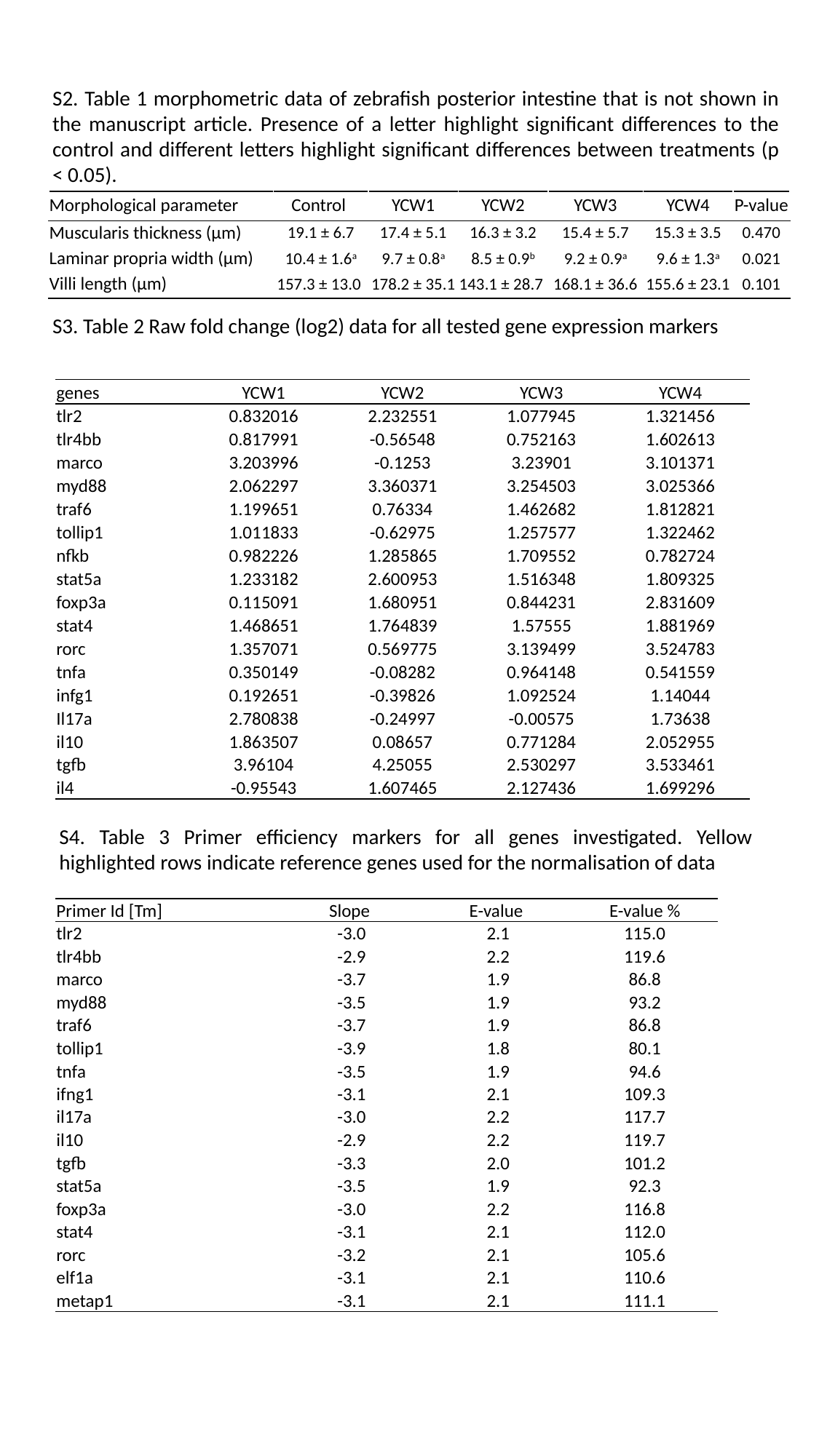

S2. Table 1 morphometric data of zebrafish posterior intestine that is not shown in the manuscript article. Presence of a letter highlight significant differences to the control and different letters highlight significant differences between treatments (p < 0.05).
| Morphological parameter | Control | YCW1 | YCW2 | YCW3 | YCW4 | P-value |
| --- | --- | --- | --- | --- | --- | --- |
| Muscularis thickness (µm) | 19.1 ± 6.7 | 17.4 ± 5.1 | 16.3 ± 3.2 | 15.4 ± 5.7 | 15.3 ± 3.5 | 0.470 |
| Laminar propria width (µm) | 10.4 ± 1.6a | 9.7 ± 0.8a | 8.5 ± 0.9b | 9.2 ± 0.9a | 9.6 ± 1.3a | 0.021 |
| Villi length (µm) | 157.3 ± 13.0 | 178.2 ± 35.1 | 143.1 ± 28.7 | 168.1 ± 36.6 | 155.6 ± 23.1 | 0.101 |
S3. Table 2 Raw fold change (log2) data for all tested gene expression markers
| genes | YCW1 | YCW2 | YCW3 | YCW4 |
| --- | --- | --- | --- | --- |
| tlr2 | 0.832016 | 2.232551 | 1.077945 | 1.321456 |
| tlr4bb | 0.817991 | -0.56548 | 0.752163 | 1.602613 |
| marco | 3.203996 | -0.1253 | 3.23901 | 3.101371 |
| myd88 | 2.062297 | 3.360371 | 3.254503 | 3.025366 |
| traf6 | 1.199651 | 0.76334 | 1.462682 | 1.812821 |
| tollip1 | 1.011833 | -0.62975 | 1.257577 | 1.322462 |
| nfkb | 0.982226 | 1.285865 | 1.709552 | 0.782724 |
| stat5a | 1.233182 | 2.600953 | 1.516348 | 1.809325 |
| foxp3a | 0.115091 | 1.680951 | 0.844231 | 2.831609 |
| stat4 | 1.468651 | 1.764839 | 1.57555 | 1.881969 |
| rorc | 1.357071 | 0.569775 | 3.139499 | 3.524783 |
| tnfa | 0.350149 | -0.08282 | 0.964148 | 0.541559 |
| infg1 | 0.192651 | -0.39826 | 1.092524 | 1.14044 |
| Il17a | 2.780838 | -0.24997 | -0.00575 | 1.73638 |
| il10 | 1.863507 | 0.08657 | 0.771284 | 2.052955 |
| tgfb | 3.96104 | 4.25055 | 2.530297 | 3.533461 |
| il4 | -0.95543 | 1.607465 | 2.127436 | 1.699296 |
S4. Table 3 Primer efficiency markers for all genes investigated. Yellow highlighted rows indicate reference genes used for the normalisation of data
| Primer Id [Tm] | Slope | E-value | E-value % |
| --- | --- | --- | --- |
| tlr2 | -3.0 | 2.1 | 115.0 |
| tlr4bb | -2.9 | 2.2 | 119.6 |
| marco | -3.7 | 1.9 | 86.8 |
| myd88 | -3.5 | 1.9 | 93.2 |
| traf6 | -3.7 | 1.9 | 86.8 |
| tollip1 | -3.9 | 1.8 | 80.1 |
| tnfa | -3.5 | 1.9 | 94.6 |
| ifng1 | -3.1 | 2.1 | 109.3 |
| il17a | -3.0 | 2.2 | 117.7 |
| il10 | -2.9 | 2.2 | 119.7 |
| tgfb | -3.3 | 2.0 | 101.2 |
| stat5a | -3.5 | 1.9 | 92.3 |
| foxp3a | -3.0 | 2.2 | 116.8 |
| stat4 | -3.1 | 2.1 | 112.0 |
| rorc | -3.2 | 2.1 | 105.6 |
| elf1a | -3.1 | 2.1 | 110.6 |
| metap1 | -3.1 | 2.1 | 111.1 |
